# Optimal mode for delivery of seasonal malaria chemoprevention in Ouelessebougou, Mali: A cluster randomized trial

**DOI:** 10.1371/journal.pone.0193296

**Published:** 2018-03-05

**Authors:** Amadou Barry, Djibrilla Issiaka, Tiangoua Traore, Almahamoudou Mahamar, Boubacar Diarra, Issaka Sagara, Diakalia Kone, Ogobara K. Doumbo, Patrick Duffy, Michal Fried, Alassane Dicko

**Affiliations:** 1 Malaria Research and Training Center, Faculty of Medicine, Pharmacy and Dentistry, University of Sciences Techniques and Technologies of Bamako, Bamako, Mali; 2 Reference Health Center of the district of Ouelessebougou, Ouelessebougou, Mali; 3 National Malaria Control Program, Bamako, Mali; 4 Laboratory of Malaria Immunology and Vaccinology, NIAID/NIH, Rockville, MD, United States of America; Université Pierre et Marie Curie, FRANCE

## Abstract

**Background:**

Seasonal malaria chemoprevention (SMC), the administration of complete therapeutic courses of antimalarials to children aged 3–59 months during the malaria transmission season, is a new strategy recommended by the World Health Organization (WHO) for malaria control in Sahelian countries such as Mali with seasonal transmission. The strategy is a highly cost-effective approach to reduce malaria burden in these areas. Despite the substantial benefits of SMC on malaria infection and disease, the optimal approach to deliver SMC remains to be determined. While fixed-point delivery (FPD) and non-directly observed treatment (NDOT) by community health workers are logistically attractive, these need to be evaluated and compared to other modes of delivery for maximal coverage.

**Methods:**

To determine the optimal mode fixed-point (FPD) vs door-to-door delivery (DDD); directly observed treatment (DOT) vs. non- directly observed treatment (NDOT)), 31 villages in four health sub-districts were randomized to receive three rounds of SMC with Sulfadoxine-pyrimethamine plus Amodiaquine (SP+AQ) at monthly intervals using one of the following methods: FPD+DOT; FPD+NDOT; DDD+DOT; DDD+NDOT. The primary endpoint was SMC coverage assessed by cross-sectional survey of 2,035 children at the end of intervention period.

**Results:**

Coverage defined as the proportion of children who received all three days of SMC treatment during the three monthly rounds based information collected by interview (primary endpoint) was significantly higher in children who received SMC using DDD 74% (95% CI 69% - 80%) compared to FPD 60% (95% CI 50% - 70%); p = 0.009. It was similar in children who received SMC using DOT or NDOT 65%, (95% CI 55% - 76%) versus 68% (95% CI 57% - 79%); p = 0.72.

**Conclusions:**

In summary, door-to-door delivery of SMC provides better coverage than FPD. Directly observed therapy, which requires more time and resources, did not improve coverage with SMC.

**Trial registration:**

ClinicalTrials.gov NCT02646410

## Introduction

Despite improved access to effective antimalarial treatments with artesunate-based combination therapy, and improved access and use of insecticide-treated nets (ITNs) and other malaria interventions, malaria continues to be a major cause of morbidity and mortality. An estimated 214 million new cases occurred in 2015 globally, causing 438 000 deaths. Sub-Saharan Africa is disproportionately affected, suffering 90% of global malaria deaths with more than two thirds occurring in children under 5 years of age [1]. In the absence of a vaccine, simple and effective control strategies are urgently needed to reduce the malaria burden in sub-Saharan Africa.

We have shown that Seasonal Malaria Chemoprevention (SMC), previously known as Intermittent Preventive Treatment of Malaria in children, reduces malaria infection and disease by more than 80% in Malian children [2]. Evidence from this study and others have prompted WHO to approve SMC as a policy for Sahelian countries in March 2012 [3]. SMC consists of the administration of full treatment courses of antimalarial drugs during the malaria season to prevent malarial illness, with the objective of maintaining therapeutic antimalarial drug concentrations in the blood throughout the period of greatest malarial risk. The WHO recommendation states that a complete treatment course of Sulfadoxine-pyrimethamine plus Amodiaquine (SP+AQ) should be given to children aged between 3 and 59 months at monthly intervals, beginning at the start of the transmission season, to a maximum of four doses during the malaria transmission season [3]. The strategy has been shown to be highly effective, safe and cost-effective for preventing malaria in children under 5 years of age in areas with highly seasonal malaria transmission such as the Sahel region in Africa [3].

Although several potential approaches can be used to implement the strategy, there is insufficient evidence to recommend a standard strategy to deliver SMC [4]. The delivery of SMC through community health workers was shown to be a potentially promising approach to implement the strategy of SMC [5]. Two studies have compared the delivery of SMC through community health workers and health facilities in Ghana and The Gambia [6; 7]. In the Gambia study, higher coverage of SMC was found when SMC was delivered through community health workers (CHW) at a fixed point compared to staff attached to Reproductive and Child Health trekking clinics [7], while in Ghana similar coverage of SMC was achieved when SMC was delivered by CHW or by staff of health facilities (69% community delivery and 66% facility delivery). In these studies, children were invited to a fixed point in the village for drug administration at each round. No study of our knowledge has compared the benefit of door to door delivery to fixed point delivery or directly observed treatment compared to non directly observed treament, when the strategy is delivered by the community health workers. Our study aimed to assess this.

## Material and methods

### Study site

The study was conducted in the district of Ouelessebougou. Ouelessebougou is located about 80 km south from Bamako, the capital city of Mali, It contains the district health center, and a Clinical Research Center of the Malaria Research and Training Center (MRTC) hosted within the community health center. In 2014 the district encompassed 14 health sub-districts. Malaria transmission is highly seasonal with an incidence rate of about 2 episodes/child/year and parasite prevalence of 7.5% in children under 5 years of age [2]. This study was carried out in four health sub-districts randomly selected from a total of 14 sub-districts. The total population of these four selected sub-districts organized into 31 villages was estimated to be 41,652 in 2014 with children under 5 years representing 18.3%.

### Study population

The target population was children aged 3–59 months of age in the district of Ouelessebougou in Koulikoro region, Mali. The study was carried out in 31 villages of the four randomly selected health sub-districts of Ouelessebougou.

### Design

The study was an open cluster randomized controlled trial with two by two factorial design. Thirty one villages of four sub-districts of Ouelessebougou were randomized at 1:1 ratio to receive either door–to door delivery (DDD) or fixed point distribution (FPD) of SMC. The same villages were also randomized to receive SMC as directly observed therapy (DOT) or non-directly observed therapy (NDOT). The unit of randomization was the village and all eligible children in a village were allocated to the same study arm (DDD+ DOT, DDD+NDOT,FPD+ DOT or FPD + NDOT).

### Randomization

The randomization was computer-generated using a simple randomization method (a single sequence of random assignments) by the study statistician based at the National Institutes of Health in US, using the list of the villages in the four selected sub-districts. The list of randomization was sent to the study team for implementation. The investigator who oversaw the coverage survey and sample collection was not blinded.

### Screening and enrollment

After discussions with the Ministry of Health and district health officials, study investigators accompanied by one member of district health and the president of the community health association, visited each of study villages in the four selected study sub-districts, to explain the purpose and procedures and seek community permission during meetings with heads of families and the village leaders. After community permission was obtained, a census of the target population (children under 5 years of age) in the 31 villages was carried out between June 22nd and July 8th, 2014 prior to starting the intervention. Demographic information such as date of birth, gender and bednet usage was collected during the census.

Screening and administration of the first round of SMC occurred from August 7^th^ to 26^th^, 2014. Prior to the scheduled day of the intervention in each village, parents were informed to make the children in the target age group available at home in villages randomized to door-to-door delivery or to bring the children to a specified fixed location in the villages randomized to fixed point delivery.

### Eligibility criteria

Children of either sex aged 3–59 months of age at the start of each period of drug administration in August 2014, were eligible for inclusion in the trial, provided that parental consent was obtained. Exclusion criteria were as limited as possible to make the results of the trial broadly applicable, and included severe chronic illness, a known allergy to one of the study drugs, or known HIV-positive subjects using Cotrimoxazole. Children with acute clinical malaria at the time of SMC drug administration were included in the study but were treated with Artemisinin Combination Treatment and did not receive SMC drugs during that round per WHO recommendation.

### Intervention

Eligible children received a course of SP+AQ on three occasions at monthly intervals during the peak of the malaria transmission season, starting in August 2014. During each round, children aged 3–11 months received 75 mg of AQ given once daily for 3 days plus a single dose of 250/12.5 mg of SP, while children aged 12–59 months received 150 mg AQ base given once daily for 3 days and a single dose of 500/25 mg of SP. The single dose of SP was given only on the first day, at the same time as the first dose of AQ. Children were observed for 30 minutes after drug administration, and were re-administered after vomiting during this period.

In villages assigned to DDD, CHWs visited each compound to administer SMC drugs. In the villages assigned to FPD, children received SMC at a central fixed point in the village. Administration of three days treatments at each round was done by CHWs for children assigned to DOT, while for children assigned to NDOT, the first day treatment was given by CHWs and treatments for second and third days were given by the caregiver at home for each round.

### Outcomes and assessment of the outcomes

The primary endpoint of the trial was the coverage rate of SMC defined as the proportion of children who received all three days of SMC treatment during the three monthly rounds determined by interview. Secondary endpoints included proportion of children who received at least one round of SMC, proportion of children who received at least two rounds of SMC, proportion of children who did not receive SMC, proportion of children who received the first day treatment of SMC per round based on the interview, and proportion of children who received the first day treatment of SMC per round based on information from the SMC card. These were assessed at the cross-sectional survey at the end of the transmission season in a randomly selected sample of the children, through interviews of the caregivers and based on information in the child SMC administration card at the end of the intervention period in November 2014. Children surveyed were selected using a simple random sampling method (from the list of children enrolled in the study). The survey was done by staff not involved with the SMC implementation and not aware of the design and research question to reduce the risk of bias and trained using a standard operating procedures designed for this survey (S1 Text). During this survey, opinions of the parent/caregiver about SMC were also collected by interview.

### Ethics

The study protocol and consent forms were reviewed and approved by the Ethical Committee of the Faculty of Medicine, Pharmacy and Dentistry of the University of Bamako (United States of America Department of Health and Human Services/ Office for Human Research Protections (US, DHHS/OHRP) Federal Wide Assurance #: FWA00001769). The approval letter was issued on June 17, 2014. Community consent was obtained for each village prior to the start of the study. Individual, written, and informed consent was obtained from a parent or guardian of each child prior to screening and enrolment in the study. The trial was registered in December 2015. The delay was due to an oversight as this was omitted in the study specific activities described in the protocol. The authors confirm that all ongoing and related activities for this study are registered.

### Sample size

The sample size was calculated using standard methods for comparison of two proportion in Stata version 12.0 (StataCorp LLC, College Station, Texas, USA). Assuming 60% coverage of SMC by the combination of least optimal methods (delivery and observation methods), 300 children/arm were needed to detect 10% increase in coverage with at least 85% power correcting for multiple tests and using two tailed Fisher's exact test. To account for cluster sampling using "sampclus" option with about 10 clusters per arm, interclass correlation of 0.01 (based on a previous trial showing vaccine coverage [8], and 5% missing data, 446 children per group (total of 1784 children) were needed.

### Statistical analysis

Data were analyzed to determine the optimal mode of delivery by comparing primary and secondary endpoints per treatment arm. Data were entered and verified using DataFax and exported to Stata (Houston, Texas, USA). An intention-to-treat analysis was used. Primary and secondary endpoints were compared between arms with 95% confidence intervals estimated using survey (svy) commands in Sata (www.stata.com/manuals13/svy.pdf), to take into account the fact that observations may be correlated within village (cluster), but would be independent between villages. Proportions were compared using F statistic, a conversion of Pearson chi squared statistic corrected for the survey design with the second-order correction of Rao and Scott [9], means were compared using adjusted Wald test.

## Results

### Recruitment and participants' characteristics

**Fig 1:**
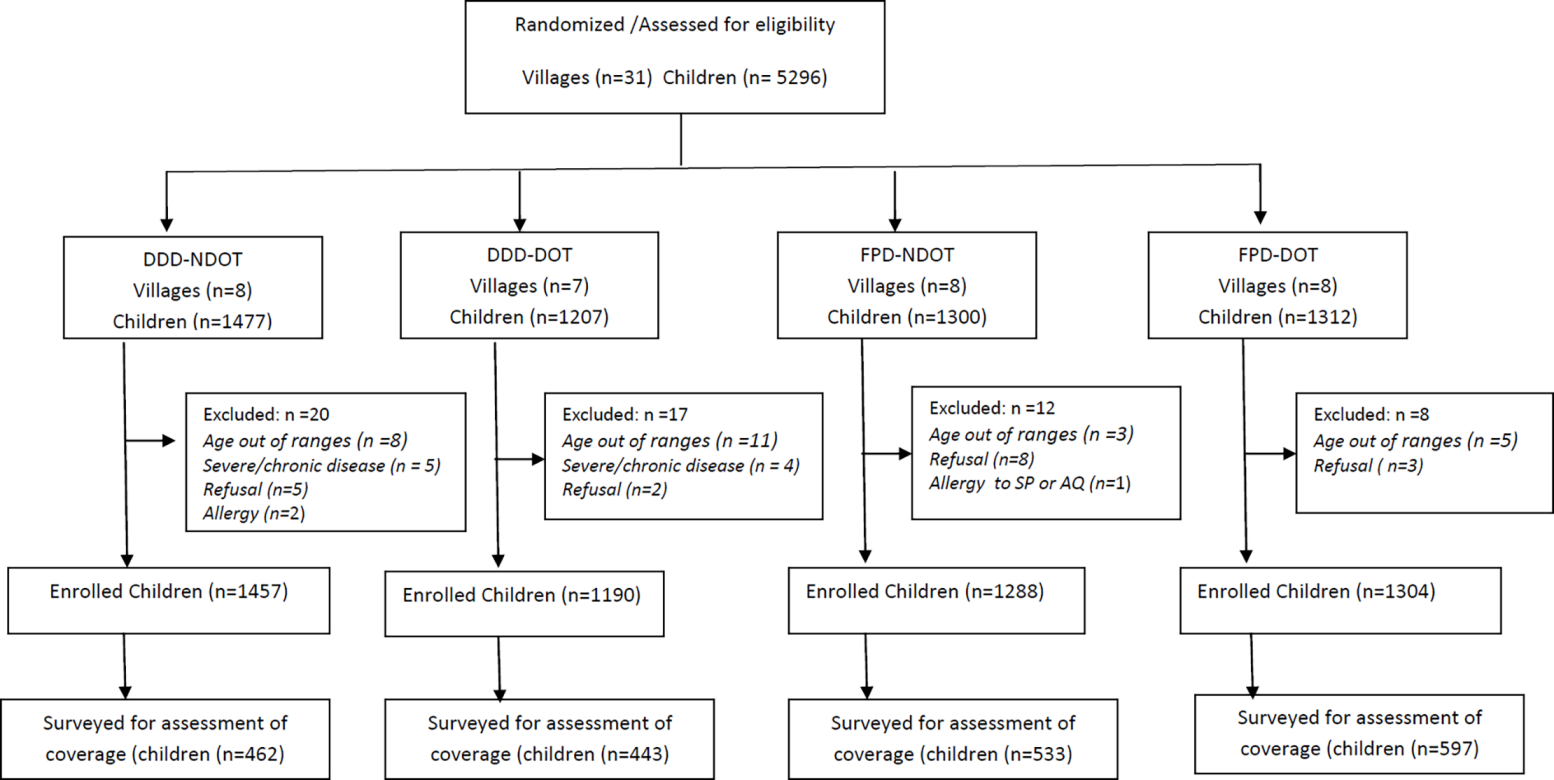
Flow diagram.

A total of 5,296 children in the 31 villages were randomized (Fig 1) and 2,035 children were surveyed three to four weeks after the last round of SMC. Participants' characteristics are summarized in Table 1. There were no differences in age and gender distribution between participants according to the type of delivery of SMC (DDD or FPD) or type of observation of the treatment (DOT or NDOT).

**Table 1:** Demographic characteristics by method of delivery and the type of observation of the treatment.

		DDD	FDP	DOT	NDOT
		(n = 905)	(n = 1130)	(n = 1040)	(n = 995)
**Age in months**					
	Mean (sd*)	33.9 (15.6)	34.6 (16.3)	34.2(16.0)	34.3 (16.0)
	Median	33	34	33	33
**Gender (%)**					
	Male	49.7	48.3	49.8	48
**Person interviewed (%)**					
	Mother	78.6	77.6	75.6	80.6
	Father	3.3	4.2	4.5	3
	Others	18.1	18.2	19.9	16.4

*sd is the standard deviation

The child’s mother was the respondent during the interview for 75% or more of the children surveyed, and this did not differ between groups.

### Coverage of SMC

Coverage of SMC according to the method of delivery for each SMC round and for all three rounds of SMC is presented in Table 2. Full coverage of SMC defined as the proportion of children who received the three days of SMC treatments for three rounds was higher using the DDD method compared to the FPD method (74.1% versus 60.1%, p = 0.009). Coverage of SMC at each of the three rounds was also significantly higher with the DDD method compared to the FPD method (Table 2). The inter cluster correlation coefficient was 0.09 (95% CI 0.03–0.14).

**Table 2:** Coverage of SMC defined as the proportion of children who received all three days of SMC treatment according to the method of delivery (door to door versus fixed point) in 2014.

	DDD		FPD		
	(n = 1040)		(n = 995)		
	%	95% CI	%	95% CI	p
**Round 1**	87.6	83.1–92.1	76.8	69.4–84.1	0.007
**Round 2**	90.2	87.4–92.9	83.0	77.0–89.0	0.014
**Round 3**	88.7	85.2–92.3	78.7	68.0–89.4	0.029
**Rounds 1, 2, 3**	74.1	68.5–79.8	60.1	50.2–70.0	0.009

The proportions of children who did not receive any round of SMC and the proportions of children who received at least one or two rounds of SMC according to the method of delivery, are presented in Table 3. The proportion of children who did not receive any round of SMC was significantly lower (1.5%) in children who received SMC door to door compared to those who received SMC at a fixed point (5.8%, p < 0.01), while the proportion of children who received at least two rounds of SMC was significantly higher with the DDD method compared to the FPD method (93.9% versus 84.2%, p <0.01) (Table 3).

**Table 3:** Proportions of children who did not receive any round of SMC, who received at least one round, and who received at least two rounds, according to the method of delivery (door to door versus fixed point).

	DDD		FPD		
	(n = 905)		(n = 1130)		
	%	95% CI	%	95% CI	p
**No SMC received**	1.5	0.6–2.5	5.8	1.8–9.9	0.002
**> = 1 rounds of SMC**	98.5	97.5–99.4	94.2	90.1–98.2	0.002
**> = 2 rounds of SMC**	93.9	91.9–95.9	84.2	76.5–91.8	0.001

The proportions of children who received the three days treatments of SMC according to the method of observation of the treatment are presented in Table 4. Proportions were similar in children who received SMC as DOT or as NDOT (65.1% versus 67.6%, p = 0.72). Proportions of children who received the three days treatments of SMC were also similar between DOT and NDOT arms at each of the three SMC rounds (Table 4).

**Table 4:** Coverage of SMC defined as the proportion of children who received all three days SMC treatments according to the method of observation of the treatment, directly observed treatment versus not directly observed treatment.

	DOT		NDOT		
	(n = 1040)		(n = 995)		
	%	95% CI	%	95% CI	p
**Round 1**	78.9	70.2–87.7	84.3	78.6–90.1	0.26
**Round 2**	85.2	78.7–91.7	87.2	81.8–92.7	0.60
**Round 3**	84.3	76.3–92.4	81.9	70.3–93.4	0.70
**Rounds 1, 2, 3**	65.1	54.7–75.5	67.6	56.5–78.8	0.72

The proportions of children who did not receive any round of SMC, who received at least one, and who received at least two rounds of SMC according to the method of observation are presented in Table 5. The proportion of children who did not receive any round of SMC was similar in DOT and NDOT arms (4.7% versus 3.1%, p = 0.66). The proportion of children who received at least two rounds of SMC was also similar in DOT and NDOT arms (88.1% versus 88.9% p = 0.86).

**Table 5:** Proportions of children who received none, at least one, or at least two rounds of SMC according to the method of observation of the treatment (directly observed treatment versus not directly observed treatment).

	DOT		NDOT		
	(n = 1040)		(n = 995)		
	%	95% CI	%	95% CI	p
**No SMC received**	4.7	0.1–9.3	3.1	0.5–5.7	0.49
**> = 1 rounds of SMC**	95.3	90.7–99.9	96.9	94.3–99.4	0.49
**> = 2 rounds of SMC**	88.1	80.2–95.9	88.9	81.7–96.2	0.86

### Mother opinions about the SMC

Mothers were asked to grade SMC as bad, good or very good. Overall of the 1588 mothers who responded, 7.9% (95% CI 4.9% to 10.9%) of them had no opinion about SMC. The proportion of mothers who had no opinion was higher in villages randomized for FPD (10.3%, 95% CI 5.9% to 14.7%) compared to villages randomized for DDD (5.1%, 95% CI 5.9% to 14.7%); p = 0.03. There was no difference in proportion of mothers with no opinion between villages who received SMC as DOT (8.8%, 95% CI 3.8% - 13.8) or NDOT (7.1% 95% CI 3.2% - 11.0), p = 0.58. Among mothers who had an opinion about SMC, 99.7% (95% CI 99.4% - 100%) reported that SMC was good or very good and only 0.3% (95% CI 0.0–0.6%) responded that SMC was bad. The main reason for negative opinions was that the child felt sick after receiving SMC drugs (4/5). There were no significant differences in mother’s opinion based on the delivery method of SMC and treatment observation (Table 6).

**Table 6:** Mother's opinion on SMC according to the method of delivery (door to door versus fixed point) and the method of observation of the treatment (directly observed treatment versus not directly observed treatment).

		DDD		FPD			DOT		NDOT		
		(n = 683)		(n = 806)			(n = 724)		(n = 765)		
		%	95% CI	%	95% CI	p	%	95% CI	%	95% CI	p
**Mother opinion**						0.12					0.27
	**Bad**	0.59	-0.01–1.20	0.12	-0.15–0.41		0.28	-0.10–0.66	0.4	-0.19–0.99	
	**Good**	29.72	20.22–39.03	41.2	28.9–53.21		31.63	20.76–42.28	40	29.37–50.36	
** **	**Very Good**	69.69	60.19–79.36	58.68	46.59–71.1		68.09	57.62–78.78	59.7	49.04–70.42	

## Discussion

In this study, coverage of SMC defined as the proportion of children who received the three days SMC treatments of the three rounds based on the information collected by interview was significantly higher with the DDD method compared to the FPD method. Proportions of children who received at least one or at least two rounds of SMC were significantly higher in the DDD arm compared to the FPD arm.

While previous studies compared SMC delivery by CHWs versus other health staff [7, 6], to our knowledge this is the first study to compare DDD and FPD methods for SMC. In a qualitative study in Katsina state, northern Nigeria, views were divergent as to which of the two methods is most effective [8]. Some respondents favored a FPD system because it was perceived as an “easier” delivery option while others considered a DDD approach to be preferable because of the potential to reach and engage with a higher number of targeted children resulting in higher coverage [10]. Our findings support the view that DDD will result in higher coverage. Although, differences may exist between countries or even within a country that can affect the coverage of malaria interventions, optimal delivery method varies with the type of intervention. For example, a higher ITN ownership was achieved using FPD compared to DDD in Senegal [11], while spray coverage was much higher with DDD compared to a mobile messaging approach in Koulikoro, Mali [12].

During earlier SMC clinical efficacy trials, drugs were administered under DOT for the three days, but this approach is difficult to maintain in implementation. For this reason only, first day treatment was directly observed in countries where SMC has been implemented, and there have been concerns that parents might not administer the second and third day treatment of SMC. This study found that DOT on the second and third days of treatment did not improve the coverage. This is consistent with the results from an observational study in the Kita district of Mali that found that > 93% of caregivers give the second and third day treatment at home [13]. Other studies have also reported good adherence to all three days of Artemether-Lumefantrine when given by the caregiver [14] and to the second and third day treatments under DOT in Mbarara, Uganda [15].

The coverage of SMC using FPD in this study is consistent with that reported in other studies in Mali [11], in the Gambia [7], and in Ghana [6]. Coverage of individual rounds of SMC varied between 84% and 89% in children who received SMC, with 76% of children who received all three rounds of SMC using DDD method while a consistently greater than 80% coverage over three years was reported in a large stepwise study in Senegal [16].

In Mali, SMC has been delivered mainly through fixed points. Our study supports the use of DDD to improve the coverage. While the cost of the delivery of SMC was not assessed in this study, in the Senegalese study that used DDD it was estimated at 0.5 USD/$ per child per round [16] and in Kita, Mali where SMC was delivered using the FPD strategy, the cost was estimated to 0.76 USD per child per round, suggesting that DDD may be less expensive than fixed point delivery.

Not surprisingly, parents’ opinion was positive about SMC with 99.7% reporting that SMC was good or very good. Similar results were reported in Kita [13] suggesting that the strategy is widely appreciated in Mali. As expected, only a very small proportion (<1%) was not happy about the strategy due to side effects associated with these drugs. The proportion of mothers who had no opinion about SMC was lower in the DDD arm compared to the FPD arm, reflecting the higher SMC coverage in the DDD arm.

Strengths of this study include the cluster randomized design and the random selection of the children to ensure compatibility and reduce the potential for selection bias. The factorial design allowed the comparison of the two methods of delivery and the two methods of the observation as well as the combination of these methods. To reduce observational bias, the coverage survey data were collected by staff not involved with the SMC implementation and not aware of the design and research question. This study was conducted in rural areas where live most of the population in sahalian countries and where the malaria burden is higher and can be generalized to this population. The results may be less generalizable to large urban areas where parents and children may not be at home during the day and where parents may be more willing and available to bring their children at fixed points for the intervention. Limitations of the study include the potential for recall bias that could not be excluded despite the fact that the coverage survey was performed only ~three to four weeks post SMC rounds, but this should be similar between the treatment arms. A comparison of the cost per child per round of these two delivery methods would have been useful and is being considered in post-study analyses.

In summary, door-to-door delivery of SMC provides better coverage than fixed point delivery. Directly observed therapy, which requires more time and resources, did not improve coverage with SMC. The highest coverage was obtained with DDD combined to NDOT.

## Supporting information

S1 Text Standard operating procedures designed for coverage survey. (PDF)

S2 Text Study protocol. (PDF)

S3 Text CONSORT checklist. (DOCX)

S1 Table Dataset. (ZIP)

## Abbreviations

AQ: amodiaquine
CHW: Community Health workers
DDD: door-to-door delivery
DOT: directly observed therapy
NDOT: non-directly observed therapy
FPD: fixed-point delivery
IC: Confidence intervals
ITN: insecticide-impregnated bed nets
MRTC: Malaria Research and Training Center
SMC: seasonal malaria chemoprevention
SP: sulfadoxine-pyrimethamine
USAID: United States of America Agency for International Development
US, DHHS/OHRP: United States of America Department of Health and Human Services/ Office for Human Research Protections
WHO: World Health Organization
