# Supplementary material for: Optimal mode for delivery of seasonal malaria chemoprevention in Ouelessebougou, Mali: A cluster randomized trial
Source: PLoS One. 2018 Mar 5;13(3):e0193296. doi: 10.1371/journal.pone.0193296 (PMC5837084; doi:10.1371/journal.pone.0193296)
Supplement: S1 Text — (PDF) [file pone.0193296.s001.pdf]

MALARIA RESEARCH & TRAINING CENTER BAMAKO, MALI	Mode Opératoire Normalisé (MON)<br>Optimisation de la mise en œuvre de la Chimio prévention du Paludisme Saisonnier (CPS) et ses effets sur l'acquisition de l'immunité anti palustre.

SOP COUVERTURE DE LA CPS CHEZ LES ENFANTS DE 3 MOIS A 5 ANS

Objectif :

- Enquête auprès des ménages sur la base des cartes d'administration données aucours passages. Dans les quatre aires de santé
- Collecte des données d'administration des trois et quatre passages et raisons de non administration.

Logistique nécessaires pour aller sur le terrain:

CRF de couverture, stylo et crayons, registre des enfants recensés, la liste des concessions avec le nom des chefs de concessions.

Deux véhicules et chauffeurs, plus le personnel infirmier qualifié

Collecte des données de Couverture CPS :

Faire la répartition des villages entre les équipes (les concessions étant numérotées).

Une fois la répartition des équipes est faite, tôt le matin, toutes les équipes sont redéployées sur le terrain.

Dans chaque village la permission communautaire est demandée et acquise avant le déroulement de l'enquête.

Une foi dans une concession :

- Au niveau de chaque concession la permission du chef de ménage doit être demandée tout en expliquant l'objectif de l'enquête avant de commencer.
- Identifier le ménage et demander les cartes d'administration des enfants.

- Une fois la carte est acquise, commencer à poser les questions à la mère ou au parent de l'enfant si il a reçu ou pas le médicament tout en vérifiant l'exactitude des informations sur la carte avant de de remplir les cases du CRF.
- Les questions doivent être posées pour tous les rounds et les raison de non administration sont également à porter sur le CRF.
- Commencer à remplir l'ID et les informations sur l'âge, le sexe et la personne interviewée
- Pour la question 3 : Si l'enfant a reçu la CPS, vérifier sur la carte et avant de cocher la case oui et passer directement à la question 4.
- Si l'enfant n'a pas reçu la CPS pour ce round cocher la case non et indique la raison de non administration.
- La question4 est à remplir uniquement pour les enfants qui ont reçu la CPS.
- Pour la question 4 les trois cases doivent être remplie en fonction des informations du jour1 ; du jour2 et du jour3 du round tout en se référant aux codes indiqué à cet effet.
- Une fois les informations sur la question 4, 5 et 6 sont complété, passer à la question 7 pour demander le tuteur de l'enfant si l'administration du médicament a été facile à domicile. Si oui passez à la question 8 ; et si non donner les raisons.
- Pour la question 8 demandez toujours les parents de leur perception sur la qualité de la stratégie avant de cocher les cases de la question 8.
- Posez toujours la question 8.1 et noter toutes les informations apportées par les tuteurs des enfants avant de passer à un autre enfant du ménage.
- Avant de passer à un autre enfant du ménage, assurez-vous toutes informations sur la carte sont reportées sur le CRF avant de passer à un autre enfant du même ménage.

Fait à Ouelessebougou le 4 Novembre 2014
