# Supplementary material for: Optimal mode for delivery of seasonal malaria chemoprevention in Ouelessebougou, Mali: A cluster randomized trial
Source: PLoS One. 2018 Mar 5;13(3):e0193296. doi: 10.1371/journal.pone.0193296 (PMC5837084; doi:10.1371/journal.pone.0193296)
Supplement: S2 Text — (PDF) [file pone.0193296.s002.pdf]

PROTOCOL OF PEER –PROPOSAL

Title: Optimization of SMC delivery and its effects on the acquisition of malaria immunity.

Country of Implementation: Mali

Study Duration: 36 months

1. PROTOCOL SUMMARY (Limit to 1-2 pages)

Background (Issue being addressed):

Based in part on the pivotal studies we conducted in Mali, SMC was approved by WHO as a policy for malaria control in countries with seasonal malaria transmission such as Mali in March 2012. The strategy is a highly cost-effective approach to reduce childhood mortality in these areas. Despite the huge benefit of the SMC on malaria infection and disease, the optimal approach to deliver SMC remains to be determinedand there is no data on the long term effect of this strategy on the development of immunity to malaria. While fixed-point delivery (FPD) combined with non directly observed treatment (NDOT) by community health workers is attractive for the SMC implementation, it is need to be evaluated and compared to other mode of delivery. Using appropriate study designs, this project will compare differ approaches to implement SMC, and will also assess the effect of one, two and three years of SMC on malaria immunity using state of art immunology techniques and novel antigens.

Research Questions/Goals and Specific Objectives:

The objectives are to identify the most effective method to deliver SMC, and to obtain information on the long term impact of SMC on malaria immunity. Specifically, i) to determine the optimal mode (fixed-point (FPD) vs door-to-door delivery (DDD); directly observed treatment (DOT) vs. non-DOT (NDOT)) and frequency (3 vs. 4 doses per season) of SMC delivery; ii) to compare quantitative measures of immunity in children who do and do not receive SMC over a three year period.

General Description of Study Design and Methods:

The design is a cluster-randomized trial over three years. The target population is children aged 3-59 months old living in Ouelessebougou district, Mali. In Year 1, villages in four sub-districts will be randomized into four groups (FPD+DOT; FPD+NDOT; DDD+DOT; DDD+NDOT). The optimal mode of delivery will be selected based on the SMC coverage during the first year,

and will then be implemented in villages of two additional sub-districts. Villages in these two sub-districts will be randomized in two groups. Children in the first group will received three rounds of SMC and those in the second group will receive four rounds of SMC to determine the optimal frequency of SMC based on the incidence rate of clinical malaria as measured by passive surveillance. Children in the four sub-districts selected in Year 1 will continue to receive three rounds of SMC in Year 2 using the optimal mode of delivery. In Year 3, children in the randomly selected sub-districts will received SMC by the optimal delivery system determined in Years 1 -2. Immune responses will be measured and compared between the children receiving SMC to a cohort of children not receiving SMC, to assess the impact of SMC on key antimalarial antibody responses over the three year period using cross-sectional surveys at the beginning and the end of the transmission season. Prevalence of malaria infection, anemia and molecular markers of *P. falciparum* resistance to SP and AQ will be measured during these cross-sectional surveys as secondary endpoints in randomly selected sample of the children.

Expected Outcomes (How this study will inform implementation/scale-up): The anticipated outcome of the study will be a qualified optimal approach to deliver SMC in Mali, and an expanded understanding of its impact on health outcomes and antimalarial immunity.

Schematic of Study Design:

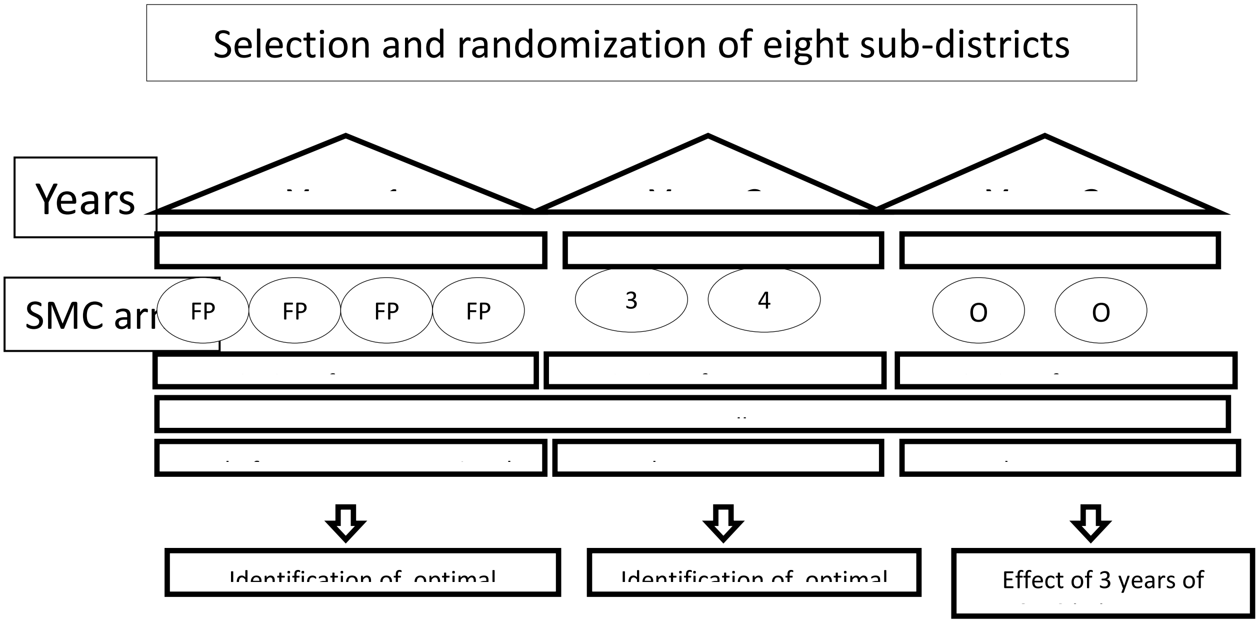

2. Background

More than 200 million people live in areas where malaria transmission is highly seasonal. Malaria remains a major cause of morbidity and mortality and is estimated to cause between 610 000−971000 deaths globally per year. Sub-Saharan Africa is disproportionately affected, suffering 91% of global malaria deaths with 88% occurring in children under 5 y of age [WHO 2012]. In absence of a vaccine, simple and effective control strategies are urgently needed to reduce the malaria burden in sub-Saharan Africa. Vector control, using insecticide-treated bednets (ITNs), insecticide-treated curtains, or indoor residual spraying (IRS), can reduce mortality and morbidity from malaria substantially [Lengeler 2004], but in high transmission settings, these interventions provide only partial protection and additional control measures are needed.

We recently showed that Seasonal Malaria Chemoprevention (SMC), previously known as Intermittent Preventive Treatment of Malaria in children, reduces malaria infection and disease by more 80% in Malian children, prompting the WHO to approve SMC as policy for Sahelian countries in March 2012 [Dicko et al 2011, WHO GMP 2012]. SMC consist of of the administration of full treatment courses of an antimalarial medicine during the malaria season to prevent malarial illness with the objective of maintaining therapeutic antimalarial drug concentrations in the blood throughout the period of greatest malarial risk. The WHO recommendation states that complete treatment course of Sulfadoxine-pyrimethamine plus Amodiaquine (SP+AQ) should be given to children aged between 3 and 59 months at monthly intervals, beginning at the start of the transmission season, to a maximum of four doses during the malaria transmission season [WHO GMP 2012].

The strategy has been shown to be highly effective, safe and cost-effective for preventing malaria in children under 5 years of age in areas with highly seasonal malaria transmission such as the Sahel region in Africa [WHO GMP 2012].

Although several potential approaches can be used to implement the strategy, there is insufficient evidence to recommend a standard strategy to deliver SMC [WHO implmentation guidelines]. Two studies have compared the delivey of SMC through community health workers and health facilities in Ghana and The Gambia [Kweku et al 2009; Bojang et al 2011]. In the Gambia, higher coverage of SMC was found when SMC is delivered through community health workers

(CHW) compared to staff attached to Reproductive and Child Health trekking clinics [Bojan et al al 2011] while in Ghana simlar coverage of SMC was when SMC is delivered by CHW or by staff of health facilities (69% community delivery and 66% facility delivery). No study has compared the benefit of directly observed treatment compared to non directly observed treament or door to door delivery to fixed point delivery when the strategy is delivered by the community health workers.

The pivotal efficacy studies in Mali, Senegal and Burkina Faso that have lead to the evaluation and the recommendation of the strategy have assesed the efficacy of two or three treatment rounds of the SMC, and there is no data to support additional benefit provided by a 4th treatment round of SMC in the WHO recommendation. In this study we will evaluate the efficacy of 4th round of the SMC treatment against clinical malaria episodes in Mali.

Rationale for This Study

Our previous studies demonstarted that SMC reduces malaria infection and disease by more than 80% in Malian children. In addition to the protective effect of the intermittenet treatment other factors may have contributed to the success of these trials such as the method of treatment delivery. WHO policy to implement SMC is based on trials conducted in Mali and other sites, however, critical factors such as the optimal method of delivery that can be implemented on a country level and will achieve a similar success rate as observed in the clinical trials remained to be determined.

This study is design to answer the primary objective, that is to identify the most effective and efficient methods to deliver SMC in Mali that is urgently needed to support SMC implementation. An optimal delivery of the strategy will make a substantial contribution toward the achievement of the goals of the US President's Malaria Initiative (PMI) and the Millenium Development Goals. The most effective method of delivery will be assessed by comparing: door–to-door delivery (DDD) or fixed point distribution (FPD) of SMC and directly observed therapy (DOT) to non-directly observed therapy (NDOT).

Another specific objective is determining the number of treatment doses. Although children participated in the trials that served as the basis to develop WHO policy received 3 treatment

doses, WHO policy suggests up to 4 treatment rounds. Taking into account the logistics associated with each treatment it is critical to determined if a 4th dose will have an additional benefit over 3 rounds of treatment.

Despite the huge benefit provided by SMC, one of the concerns is that SMC will impair the acquisition of protective immune responses, thereby increasing the risk of disease in later years. Studies conducted in Mali and other countries have compared the susceptibility to malaria among children who received the intervention over a single season and the control group during the following malaria transmission season and have found a small increase in clinical malaria among children who received the intervention [Dicko et al 2008, Dicko et al 2011, Konate et al, 20011, Wilson A et al 2011]. None of these efficacy studies have assessed the impact of SMC on immunity to malaria when given for more than one transmission season. Under conditions of long term intervention, we hypothesize that immunity to liver-stage malaria antigens (reflecting exposure to infected mosquito bites) will be similar among children receiving SMC to that of children who will not receive SMC, but immunity to blood stage antigens will be markedly decreased (reflecting protection from blood stage infection). In this proposal, children will receive intervention in a stepped wedge fashion and be followed for a period up to 3 years. We are currently studying the acquisition of immunity to malaria among children recruited at birth as part of an immuno-epidemiological (IMEP) cohort study in Ouelessebougou, where we plan to enroll up to 2000 children who will be followed up for 5 years. We will leverage this cohort of children as a control group for the proposed intervention study. This will provide insights into the acquisition of malaria immunity and may indicate whether the cessation of longterm SMC will increase risk of subsequent malaria and warrant additional control measures.

Another specific objective of this study is to determine whether SMC will increase the spread of SP and AQ resistant parasites. In our previous study that evaluated the impact of SMC during one season on resistance to SP, there was no difference in the frequency of SP resistance markers between parasites collected in treatment and non-treatment villages. It is unknown if longer implementation of SMC will accelerate the accumulation of resistant parasites, nor the status of development of resistance to AQ. Our recent evaluation of IPTp in Tanzania, an area with widespread resistance to SP, IPTp was associated with increased placental parasite density and inflammation. The current study will allow comparing the frequencies of SP and AQ resistance

markers at the beginning of the study and during each subsequent year. The success of SMC in West Africa is due to limited spread of drug resistant parasites, however, changes in parasite population need to be monitored to ensure the effectiveness of this program.

Potential Use of Findings

SMC is a new intervention approved by WHO as policy in the March 2012. The main and urgent objectives of the project are to identify the most effective method to deliver SMC, and to obtain more information on the longterm impact of SMC on malaria immunity. Mali is one of the countries supported by the US President's Malaria Initiative (PMI) implemented through USAID. The aim of PMI is to reduce malaria-related deaths by 50 percent in selected countries by achieving 85 percent coverage of proven preventive and curative interventions, in support of the National Malaria Control Program. Finding the optimal delivery method of the SMC will allow optimal deployment of the strategy through the country for higher impact on the mortality and mortality from malaria in Mali and contribute in achievement toward the millimun development goal and the PMI objectives. The project will also assess the effect of SMC on malaria immunity when given over three consective years, so that better of understdaning of the malaria immunity will be gained and appropriate measures could be taken to accompagn the implementation of the strategy if needed

The project will be carried out through local health system. Staff of the health center in selected villages will be trained to carry out the SMC interventions with support of the project team composed of MRTC, LMIV and the National Malaria Control Program. The project will collaborate with the President Malaria Initiative (PMI) of the USAID mission in Bamako. The MRTC is currently assuring the training, monitoring and evaluation of US President Malaria Initiative (PMI) program to Mali funded through USAID and the collaboration will continue through this project.

3. Objectives & Outcome Measure(s)

A. Study objectives

Overall objective

The overall objective is to identify the most effective method to deliver SMC, and to obtain more information on the long term impact of SMC on malaria immunity.

Specific objectives

1. To determine the optimal mode (fixed-point (FPD) vs door-to-door delivery (DDD); directly observed treatment (DOT) vs. non-DOT (NDOT)) and frequency (3 vs. 4 doses per season) of SMC delivery
2. To compare quantitative measures of immunity in children who do and do not receive SMC.
3. To monitor the impact of SMC on the molecular markers of the resistance of *P. faliciparum* to SP and AQ.

B. Study Outcome measure(s)

The primary outcomes are:

1. SMC coverage for the determination of the optimal mode of delivery during Year 1. SMC coverage will be defined as proportion of the children who have received the three treatments doses at each of the three rounds of SMC.

2. Incidence of the clinical malaria for the optimal frequency of SMC in Year 2. Clinical malaria will be defined as signs and symptoms suggestive of malaria with positive RDT and/or positive blood smear.

Secondary trial outcomes of the study include:

1. Parasite prevalence defined as the proportion of children with a positive malaria blood smear and parasite density mesured in Years 1, 2 and 3.
2. Prevalence of moderate anemia defined as hemoglobin concentration < 8 g/dL measured by hemoglobin analyzer.
3. Cellular and humoral antimalarial immune responses to malaria parasites.
4. Levels of cytokines and other host responses measured in sera/plasma or in immune cells.

5. Frequency of mutations at codons 51, 59, and 108 of the dhfr gene, 437 and 540 of the dhps gene, codon 76 in the *P. falciparum* chloroquine transporter gene (pfcrt), and codon 86 of the *P. falciparum* multidrug resistance gene one (pfmdr1)

4. Study Design

A. Detailed Description of Study Design

Overall design

The study will use a cluster randomized design over three years. Eight sub-districts will be randomly selected among the 13 sub-districts of Ouelessebougou but excluding the sub district of the Ouelessebougou where the IMEP cohort study is ongoing. These eight sub-districts will be randomized so that the intervention begins in four districts during the first year (Year 1), in two sub-districts in the second year (Year 2) and in two remaining sub-districts in third year (Year 3). The target population is children aged 3-59 months of age in the district of the Ouelessebougou in Koulikoro region, in Mali.

In Year 1, the implementation of SMC will occur in four randomly selected sub-districts in Ouelessebougou. Villages in these sub-districts will be randomized at 1:1 ratio to receive either a door–to door delivery (DDD) or fixed point distribution (FPD) of SMC. They will also be randomized to receive SMC as directly observed therapy (DOT) or non-directly observed therapy (NDOT). A census of the target population (children aged 3-59 months) will be carried out in all the selected villages prior to the intervention. During this census, blood samples will be collected in a randomly selected subset of study population to gather baseline data on cellular and humoral responses to malaria antigens, parasites prevalence and density on blood smears and frequency of the molecular markers of the resistance to SP and AQ at the start of the transmission season. The census list will be used to deliver the intervention and will be regularly updated. SMC will consist of the administration of full treatment course of sulfadoxine-pyrimethamine plus amodiaquine (SP+AQ) at monthly interval during the malaria transmission starting in August 2014. Children will be followed passively for the determination of the

incidence of the malaria disease (uncomplicated and severe malaria). A cross-sectional survey will be carried after the third round in November/December to determine the coverage of SMC, the prevalence of the malaria parasitemia and anemia as well as humoral and cellular immune responses in a randomly selected number of children. Data collected in year 1 will be analyzed to determine the optimal delivery method (DDD+ DOT or DDD+NDOT or FPD+ DOT or FPD + NDOT) based primarily on the coverage of SMC and secondarily on the incidence of clinical malaria. The difference in SMC coverage is expected to be reflected in the incidence of clinical malaria, and the latter will be used to support the data on coverage of SMC. Should there be no statistically significant difference between the arms in coverage and incidence of the disease, the easiest method of the delivery will be recommended for Year 2.

In Year 2, SMC will be implemented using the optimal delivery system (determined in Year 1) in two sub-districts assigned during Year 2 after a census of children under 5 years of age. Villages in these two newly selected sub-districts will be randomized to receive either three or four SMC rounds during the transmission season starting in August. Baseline data on malaria parasitemia, markers of resistance to SP and AQ and immune responses to malaria antigens will be collected at baseline prior to the intervention in these two sub districts through a cross-sectional survey. In order to assess the effect of the fourth dose of SP and AQ, children will be followed passively to measure incidence rate of clinical malaria, and a cross-sectional survey will be carried out at the end of the malaria transmission season in November/December in a randomly selected sample of children to determine the prevalence of malaria infection, anemia, and humoral and cellular immune responses. Children that participated in Year 1 will continue to receive three rounds of SMC in Year 2 using the best delivery method identified in Year 1. The choice of the optimal number of doses will be based on the incidence of clinical malaria between the two arms of children in the two newly selected districts. An absolute difference of 7.5 episodes per 100 children per year will be considered meaningful.

In Year 3, all study children in the eight selected sub-districts will receive SMC by optimal method and frequency determined in Year 1 and Year 2 and will be followed passively for clinical malaria episodes through the end of the season. Venous blood samples will be collected at the beginning (July/August) and the end of the transmission season (November/ December) in a subset of randomly selected children to assess humoral and cellular responses to malaria

antigens as well as the prevalence of malaria parasitemia among children who received SMC during one, two and three malaria transmission seasons. These outcomes will also be compared to those among children in the IMEP cohort who never received SMC, in a separate analysis taking into account the differences and similarities between the cohorts.

Detailed Description of Study Population

The study population will be children aged 3-59 months living in the districts of Ouelessebougou. The study will include up to eight sub-districts in the districts of Ouelessebougou estimated and up to 10,800 children less than five years of age.

Community consent

The objectives of the study and the method to conduct the study will be discussed with the Ministry of Health and district health officials prior to its commencement. Community approval will be sought through meetings with leaders of the study communities at the sub-district level as well as the village level. During these meetings the study procedures (including information on SMC delivery) will be explained to the leaders. There will be no special advertisement for a particular method. Parents will be informed to make the children in the target age group available at home in villages randomized to door to door delivery or to bring them to a specified fixed point in the villages randomized to fixed point delivery. Leaders will have the opportunity to ask questions and provide input on the best ways to encourage community participation. They will be asked together with the community health workers to pass the information to members of the communities.

Census and randomization

Once community consent to the trial is obtained, a household census will be conducted and all households within the study areas with children under 5 years of age will be identified. At the time of census, a preliminary screening of potentially eligible children will be undertaken. Eligible children will be identified and written informed consent will be obtained from their caretakers for their inclusion in the trial. Children entered into the trial will be assigned a unique ID number and their demographic data (date of birth and/or age, and gender) will be collected.

The census will be updated at each SMC round and potentially eligible participants (such as children who did not reach the age of three months during the previous round) will be identified and screened for eligibility and enter into the study. This updated census will collect information on migration and death of children enrolled into the study for precise estimation of person-time of follow-up.

The unit of randomization will be village and all eligible children in a village will be allocated to the same study arm (DDD+ DOT or DDD+NDOT or FPD+ DOT or FPD + NDOT) to avoid confusion in Year 1. Villages in the selected sub-districts will be randomized at 1:1 ratio to receive either a door –to-door delivery (DDD) or fixed point distribution (FPD) of SMC. They will also be randomized to receive SMC as directly observed therapy (DOT) or a non-directly observed therapy (NDOT). In Year 2, villages in the two newly randomly selected districts will be randomized into two groups to receive either three or four rounds of SMC.

Inclusion and exclusion criteria

Children of either sex aged 3-59 months of age at the start of each period of drug administration will be eligible for inclusion in the trial, provided that parental consent is obtained. Children under the age of three months on the date of the start of drug administration will be excluded that year, as SMC is not recommended for children in this age group whose risk of acquiring malaria is low. Children who reach the age of 59 months or more at the end of the first or second years of treatment will not receive SMC in subsequent years. Exclusion criteria will be as limited as possible to make the results of the trial broadly applicable and will include i) a severe, chronic illness, ii) a known allergy to one of the study drugs, iii) known HIV positive subjects using Cotrimoxazole. Children with acute clinical malaria at the time of SMC drug administration will be treated by ACT and will not be given SMC drugs during that round as per WHO recommendation. They will receive SMC drugs during subsequent cycles.

Interventions

SMC will consist of the administration of full treatment courses of sulfadoxine-pyrimethamine plus amodiaquine (SP+AQ) at monthly intervals during the peak of the malaria transmission season (August – through October or November depending on the year of the study and study arm). During each round, children aged 3–11 months will receive 75 mg of AQ given once daily

for 3 days plus a single dose of 250/12.5 mg of SP, while children aged 12–59 months will received 150 mg AQ base given once daily for 3 days and a single dose of 500/25 mg of SP. The single dose of SP will be given only on the first day, at the same time as the first dose of AQ. Children will be observed for 30 minutes after drug administration. If vomiting occurs during this period, drugs will be re-administered. If vomiting recurs this will be noted but the drugs will not be given on a third occasion. Such children will not be excluded from the trial and they will be eligible to receive drugs at the subsequent monthly treatment.

In villages assigned to door –to-door delivery (DDD), community health workers (CHW) will visit each compound to administer SMC drugs. In the villages assigned to fixed point distribution (FPD), children will receive SMC at a central fixed point in the village. The administration of the three days treatments at each round will be done by the CHW for children assigned to directly observed therapy (DOT) while for children assigned to non-directly observed therapy (NDOT), the treatment of first day will be given by the CHW and treatments for second and third days will be given by the caregiver at home for each round. SMC will be implemented using standardized field implementation guidelines developed by the WHO [WHO 2013] adapted to the situation in Mali. Monitoring and evaluation tools for SMC will be developed and integrated with the health system in Mali.

Follow-up and measurement of outcomes

Surveillance for clinical attacks of malaria will start at the time of the first administration of study drugs in August and continue until the end of the third malaria transmission (November/December 2016). Clinical episodes of malaria will be detected using a passive surveillance system. Data will be collected by health care workers including the community health workers. Clinical malaria episodes occurring in the study health facilities or in the study communities will be recorded throughout the study period in all the study villages. Children with an illness suggestive of malaria will be tested for malaria with RDT and/or with blood smear by the heath center staff and/or community health workers and treated according to the recommendations of the National Malaria Control Program. Cases of clinical malaria will be recorded on standardized case report forms. Refresher training of staff will be conducted each

year to ensure standardization in the recording of symptoms and signs that are used to make a diagnosis and categorisation of clinical malaria episodes.

Each year, at the onset and the end of malaria transmission season, a cross-sectional survey will be undertaken in a random sample of children to assess the prevalence of malaria infection, anemia and immunological parameters. Coverage of SMC will be assessed at the cross-sectional survey at the end of the transmission seasons through interview of the caregivers and based on information in the child SMC administration card. Children will be examined, and a blood sample will be obtained for measurement of including hemoglobin concentration, preparation of a blood smear for microscopy, and analysis of cellular and humoral immune responses to known (CSP, MSP1, AMA1) and novel liver and blood stage *P. falciparum* antigens.

Definitions

The following definitions will be used during the course of the study.

Clinical malaria. The primary definition requires: i) the presence of fever (axillary temperature > 37.5 ^{o}C) or a history of fever in the past 24 hours; ii) the absence of any other obvious cause of the fever, sign or symptom; iii) the presence of *Plasmodium.* Asexual parasitaemia detected by blood smear and/or by RDT. A secondary definition of malaria will be i) the presence of any sign or symptoms suggestive of malaria; ii) the absence of any other obvious cause of the sign and symptoms; iii) the presence of *Plasmodium.* asexual parasitaemia by blood smear or by RDT.

Severe malaria. Severe malaria will be defined according to the WHO criteria [WHO 2000].

Anaemia. Anaemia will be defined as Hb concentration <11 g/dl, moderate anaemia as Hb concentration < 8 g/dL and severe anaemia as Hb concentration < 5 g/dl.

Molecular markers of resistance to SP and AQ. The mutations at codons 51, 59, and 108 in the dhfr gene, 437 and 540 in the dhps gene, mutations in codon 76 the *P. falciparum* chloroquine transporter gene (pfcrt), and codon 86 of the *P. falciparum* multidrug resistance gene one (pfmdr1) will be monitored.

Laboratory Procedures

Detection of malaria. RDT will be used for the initial diagnosis of malaria and to guide treatment according the guidelines of the NMCP. Parasite prevalence during cross-sectional surveys will be determined using blood smears. Blood films collected at the same time will be read subsequently by two microscopists. Slides which are judged to be discordant for either positivity or parasite density will be read by a third reader. The final result will be the median of the two or three readings.

Measurement of haemoglobin concentration. Haemoglobin concentration will be measured colorimetrically using hemoglobin analyzer (Hemocue AB, Angelholm, Sweden).

Molecular markers of resistance to SP and AQ: Filter paper samples from children with a mono-infection of P. falciparum on blood smears will be analyzed by nested PCR for mutations at codons 51, 59, and 108 of the dhfr gene, 437 and 540 of the dhps gene, 76 of *P. falciparum* chloroquine transporter gene (pfcrt), and 86 of the P. falciparum multidrug resistance gene one (pfmdr1), according to published methods [Plowe CV et al 1995, Djimde et al 2001. Dicko et al, 2010]. Cases of mixed infection (wild type and mutant) will be categorized as mutant.

Immunologic assays will include humoral antimalarial immune response to blood stage and liver stage antigens, cellular antimalarial immune responses to liver stage antigens, levels of cytokines and other host responses (e.g., erythropoietin) measured in sera/plasma or in immune cells / immune cell subsets. Our primary immune endpoints for this study will be humoral immune responses to the well-characterized pre-erythrocytic antigen CSP, and blood stage antigens MSP1 and AMA1. All three antigens are candidate antigens for malaria vaccines, and the Laboratory for Malaria Immunology and Vaccinology developed a highly standardized assays to support preclinical and clinical development of vaccines based on these antigens. Responses to CSP reflect host exposure to the preerythrocytic parasite stages (ie, sporozoites and intrahepatocytic forms), while responses to AMA1 and MSP1 reflect exposure to the blood stage forms of the parasite.

In general, the immune mechanisms that mediate naturally acquired protection from malaria are not conclusively established. For this reason, we will survey a number of humoral and cellular immune responses as secondary or exploratory aspects of the study. For example, preerythrocytic forms of the parasite, when attenuated, can induce sterile immunity in humans, and available evidence suggests that different cellular and humoral immune responses may contribute to

protection. For secondary immunological endpoints of this study, we will survey humoral and cellular immune responses to CSP and novel preerythrocytic antigens that may have a role in protective immunity. LMIV has identified several *P. falciparum* antigens whose orthologues confer protective preerythrocytic immunity in mouse malaria models, including PFL1995c, LISP1, and SAP1.

For blood stage immunity, it is known that naturally acquired IgG antibodies can clear parasite and resolve symptoms when given as passive therapy, but the targets of these antibodies are unknown. In addition to our primary endpoints of antibody levels against AMA1 nd MSP1, we will also measure functional activity of blood stage antibodies as secondary endpoints. These include antibodies that will block parasitized red cells from binding to endothelial and other receptors, which many scientists believe should reduce parasite sequestration and therefore malaria-related disease.

Based on the results of our primary and secondary immunologic studies, and the clinical findings of the trial, we may undertake more exploratory assays. For example, if the laboratory or clinical evidence suggests that SMC modulates protective antimalarial immunity, LMIV has genome based tools, such as transcriptomic or proteomic approaches, to broadly survey host responses (plasma, serum, cellular) that may be involved in malaria infection and disease.

Study discontinuation

Study may be discontinued if there is:

1. Evidence of high level of resistance of P. falciparurum to SP and AQ determined as prevalence of dhps 540 mutation > 50% or in vivo resistance of P. falciparum resistance to SP+AQ > 10%
2. Drastic reduction of the incidence clinical malaria to less than 0.1 episode per child per year without SMC intervention.

Subjects withdrawal criteria and handling

Withdrawal criteria will include:

- Withdrawal of consent, HIV positive subjects under cotrimoxazole,
- Allergy or intolerance of SMC drugs and

- Travel outside the study villages.

For these subjects the administration of SMC drugs will be discontinued, but subjects will continue to receive routine health care services provided through the heath centers in the areas.

Quality Assurance/ Quality Control of Data and Collection

An independent GCP monitor will be appointed by the sponsor (PEER-Health or NIAID/NH) to ensure that the study is conducted according to the study protocol, GCP, ethical and regulatory requirements standards are met. The monitor will conduct an initiation visit, a close out visit and at least one additional visit each year. S/he will examine a random selection of clinical and laboratory records during each visit.

SMC implementation tools will be developed in collaboration with the district health authorities. Standard operating procedures will be developed for each of the key activities including (consent, census, SMC administration, clinical exam, diagnosis and treatment of the malaria, sample collections and processing and lab techniques. Activities will be carried out by competent and trained staff. Initial training on the study protocol, GCP and GLP will be provided to the health staff in the intervention villages, as well additional staff of the project. Refresher training will be provided at least one year. Data will be collected in standardized forms. Quality control will be performed on sample of the RDTs in a routine manner and feedback and refresher training will be provided according to the procedures of the district health center. SMC drugs will be sourced through reliable channels to ensure quality. SMC drugs, ACT for treatment of uncomplicated malaria, injectable quinine and artesunate for treatment of severe malaria, and RDT for malaria diagnosis will be provided by the USAID PMI as part of their support for SMC implementation and malaria control in Mali. Quality control of the drug will be performed by independent laboratory through USAID-PMI and CDC. Diagnosis and treatment for malaria in under -5 children is free in Mali.

Patient Safety Considerations

Risks to participants in the trial are small. The trial will use only licensed products and does not involve any new methods. Over 800,000 courses of SP and AQ have been given for SMC without any report of a drug associated severe adverse event (SAE) including when enhanced surveillance was used [Wilson et al 2011, Ndiaye et al . submitted]. Neither the less the serious adverse events will be monitored throughout the study period and any SAE related to the study

drugs will be reported to the Ethical Committee of the Faculty of Medicine, Pharmacy and Dentistry of the University of the Bamako and the within 24 hours the Principal Investigator has become aware of it.

Definitions and Procedures for recording and reporting Adverse Events (AE) and Serious Adverse Events (SAE)

An adverse event (AE) is defined as any clinical symptom or sign that occurs in study children after administration of the study drugs that may or may not have a causal relationship with the study drugs. A serious adverse event (SAE) is any clinical condition that fulfils at least one of the following criteria: results in death, is life-threatening (the child was at risk of death at the time of the adverse event), results in disability/incapacity. AE and SAE will be classified by severity, causality and outcome as described below

Severity. The severity of a clinical adverse event is to be scored according to the following scale:

- Mild: Awareness of sign or symptom, but easily tolerated.
- Moderate: Discomfort enough to cause interference with usual activity.
- Severe:Incapacitating with inability to perform usual activity.
- Life-threatening: Patients at risk of death at the time of the event.
- Death

Assessment of Causality and Outcome. The relationship between the study drugs and the occurrence of each AE/SAE will be determined based on their clinical judgment. Alternative causes, such as the natural history of the underlying diseases, concomitant therapy, other risk factors, and the temporal relationship of the event to the study drug will be considered and investigated. The relationship of an adverse event to study drug is to be assessed according to the following definitions:

- Definitely unrelated: events that had occurred prior to administration of the study drugs or events that are obviously unrelated to the study (e.g. accidental injury).
- Unlikely: There is no reasonable temporal association between the study drug and the suspected event and the event could have been produced by the child's clinical state or other concomitant medications.

- Possible: The suspected adverse event may or may not have a reasonable temporal association with the administration of study drug but the nature of the event is such that an association with the study drug cannot be ruled out. The event could be related to the child's clinical state or by concomitant medications.
- Probable: The suspected adverse event follows a reasonable temporal sequence after administration of study drugs, abates upon discontinuation of the drug, and cannot be reasonably explained by the known clinical state of the child.
- Definitely related: events that have no uncertainty in their association to the administration of study drugs.

Outcome. The outcome of each AE will be assessed according to the following classification:

• Completely recovered :	The child has fully recovered with no observable residual effects
• Not yet completely recovered :	the child's condition has improved, but still has some residual effects
• Deterioration :	The child's overall condition has worsened
• Permanent damage :	The AE has resulted in a permanent impairment
• Death :	The child died due to the AE
• Ongoing :	The AE remains the same as at onset
• Unknown :	The outcome of the AE is not known because of lost to follow-up

Disaster Recovery Plans

Study data will be regularly entered in to DataFax database that is supported and maintained by the NIAID.   As with ongoing IMEP study data will maintained by the DataFax support team at NIAID/NIH with rigorous the back-up systems. The system has the advantage of generating

automatically pdf copies of the forms that can be printed to replace the records in case of disaster.

5. Statistical Considerations and Data Analysis

A. Sample Size and Power Calculation(s)

Cross-sectional surveys. Sample size for cross-sectional surveys will be based on the SMC coverage. Assuming 60% average coverage of SMC by the combination of nonoptimal methods (pools of two groups), 300 children/arm will be needed to detect 10% increase in average coverage for two pooled groups with at least 85% power correcting for multiple tests and using two Fisher's exact tests. To account for cluster sampling with a minimum 10 clusters per arm, interclass correlation of 0.01 (based on a previous trial showing vaccine coverage (Dicko et al BMC Pub Health 2011), and 5% missing data, 446 children will be surveyed per group (total of 1784 children).

Passive survaillance.

Assuming a clinical malaria attack rate of 0.3 per child per year in "3 rounds" arm, to detect an absolute difference in attack rate of the 0.075 episode/chil/ year (corresponding to 25% reduction) in the "4 rounds" arm, with a one sided p-value of 0.025 and power of 80%, using a quasi poisson model accounting for 20 equally sized clusters per arm with intraclass correlation of 0.01 and 5% missing data we will need 1386 per arm, or 2,772 subjects total.

Immunologocal parameters. We will base our sample size calculations for immunological endpoints on the humoral immune response to AMA1. We select this response because AMA1 is highly immunogenic from among malaria antigens. Also, the intervention in this trial is directed at reducing the blood stage burden of parasites during the malaria season, and the response to AMA1 reflects exposure to blood stage forms of the parasite. In earlier serology studies of young children participating in an AMA-1 vaccine trial in Bancoumana, Mali (Sagara et al, Vaccine, 2009), we observed the peak antibody titer against AMA1 to occur near the end of the malaria season (among children receiving control vaccine), with geometric mean antibody levels of 120 antibody units (95% CI, 70-205). Antibody levels just prior to the start of the malaria season (before vaccination) were 40 (25-75) antibody units. Based on these data, 467 subjects per

group will provide 80% power, to detect a 2 fold or greater reduction in peak AMA1 antibody levels among the children receiving SMC, accounting for cluster sampling in 10 equally sized clusters with 0.01 intraclass correlation as well as 10% missing data given that samples may be inadequate or spoil during routine collection and transport.

B. Planned Interim Analyses (if applicable)

At the end of the first transmission season, data will be analyzed to determine the optimal mode of delivery by comparing primarily the coverage of SMC and secondarily the incidence rate of the clinical malaria between the four intervention arms. In addition, descriptive analysis of the baseline frequencies of the immunological parameters, and molecular markers of the resistance of *P. falciparum* to SP and AQ, will also be performed at the end of Year 1.

Data collected in Year 2 will be used to determine the optimal frequency of the SMC rounds by comparing primarily the incidence of clinical malaria and secondarily the parasite prevalence at the end of season between the 2 arms. Descriptive analysis of immunological parameters and markers of drug resistance will also be performed.

Details of the statistical methods are provided below in the final analysis plan.

C. Final Analysis Plan

Describe statistical methods that will be used to analyze data.

Data will be entered and verified using DataFax. The cleaned database will be exported to Stata (Houston Texas USA) or SAS for analysis. An intention-to-treat analysis will be used except for the immunological endpoints for which the exact number of the SMC treatments received will be considered.

SMC coverage will be determined using the information on the SMC card and from interview of parent or care giver and will be determined as the proportion of children who received all the treatments at all the SMC rounds during the transmission season. The proportion of children who received at least the first treatment dose of the SMC will be also estimated as secondary endpoints for SMC coverage. SMC coverage will be compared between arms and years of

interventions with 95% confidences intervals adjusted for cluster design (using cluster option in Stata for example). The DDD+NDOT arm will be considered as control group.

The census of the children will be updated at each round and at the end of the transmission season to allow a good estimation of person-time of follow-up. Cases of the clinical malaria cases will be recorded and the incidence rate of the clinical malaria defined as the number of clinical malaria episodes divided by total child days at risk. Children will not be considered at risk 21 days after a clinical malaria episode. Incidence rates of clinical malaria will be compared between treatment arms using generalized estimating equation to estimate the incidence rate ratio, with adjustment for age, previous SMC regimens and the lack of independence among repeated episodes in the same child.

Proportions of other secondary endpoints such as parasite prevalence, prevalence of anemia and frequency of molecular markers of resistance to SP and AQ and adverse events associated with study drugs will be estimated and compared between groups using generalized linear models with 95% confidence intervals, adjusted for cluster design and potential confounding variables such age and use of ITN.

Differences at baseline will be compared using Chi-squared or Fisher's exact tests as appropriate for categorical variables and by analysis of the variances for quantitative variables that are normally distributed. Nonparametric tests will be used for non- normally distributed data.

5. Protection of Human Subjects/Ethical Considerations

A. Ethical Standard

The study will be conducted in compliance with the protocol, current Good Clinical Practice (GCP) guidelines, and all applicable regulatory requirements.

B. Human Subjects Considerations

i. Potential Risks

Risks to participants in the trial are small. The trial will use only licensed and recommended drugs and does not involve any new methods. The risks to the participants are limited to those associated with the use of SP + AQ and blood sampling. Risks occasionally associated with

finger and heel pricks and venipuncture include pain and bruising at the site of the prick, and rarely infection. Use of SP +AQ can be associated with side effects of the drugs including allergy. Subjects with known allergy to the study drugs will be excluded. Over 800,000 courses of SP and AQ have been given for SMC without any report of a drug associated severe adverse event (SAE), including when surveillance is enhanced [Wilson et al 2011, Ndiaye et al submitted]. However, a potential hazard from the intervention is the possible impact of large scale drug administration over two or three years on the drug sensitivity of malaria parasites. The possibility that SMC with SP+AQ might induce resistance to these drugs in *P. falciparum* has been investigated in a number of trials of SMC. Selection of parasites carrying mutations which confer resistance to pyrimethamine or sulphadoxine has been demonstrated in some but not all studies [Wilson et al]. However, because the prevalence of parasitaemia in children who received SMC was substantially less than in the control group, the total number of parasites carrying resistance markers was less in children who had received SMC than in control children. SP has been used extensively in intermittent preventive treatment in pregnant women and has not be associated with an acceleration of resistance to SP in West Africa. Similarly the use of SP in pilot implementation in Mali was not associated with increased frequency of the molecular markers associated with resistance to SP (Dicko et al 2008). Furthermore, the potential risk of SMC with SP +AQ in inducing resistance of malaria parasite was reviewed carefully by the WHO Technical Expert Group and considered to be an acceptable risk in light of the major benefits conveyed by the intervention. Samples collected on filter paper will be tested for markers of the resistance to SP and AQ at baseline and two years after to monitor the impact of the sensitivity of the malaria parasites to the study drugs as per the WHO recommendations.

Conduct of the trial will not impose any additional costs on the local health services. The project will contribute to the additional costs associated related to the study and the strengthening the health system in the study areas.

ii. Potential Benefits

The benefits of participation to the study include the protection against clinical malaria conferred by SMC and improved supervision of the health care provided by the CHW and the health staff. Finding the optimal delivery method of the SMC will allow optimal deployment of the strategy through the country for higher impact on malaria morbidity and mortality in Mali and other

countries. The study will also provide insights into the acquisition of malaria immunity and may indicate whether the cessation of longterm SMC will increase risk of subsequent malaria and warrant additional control measures.

C. Institutional Review Board (IRB)

The protocol and consent forms and any subsequent amendment will be submitted for approval to Ethical Committee of the Faculty of Medicine, Pharmacy and Dentistry of the University of Bamako (US, DHHS/OHRP Federal Wide Assurance #: FWA00001769). Individual consent will be obtained before inclusion in the study.

D. Informed Consent Process

Community Consent

The previous studies conducted by the MRTC in Ouelessebougou have permitted extensive contacts with the village population that has led to the development of mutual trust and the establishment of an ongoing informed consent process. The community has become familiar with the informed consent process, including written, signed consent forms. The community informed consent process goes through the following steps:

1. Explanation and clarification to village leaders, including the village chief and elders, of the purposes, procedures, and risks to the study subjects.

2. Allow time for village leaders to communicate with community members and relay any additional questions or concerns.

3. Thorough protocol explanation to the family heads.

Individual Consent

The informed consent document will be used to explain the risks and benefits of study participation to the parent or guardian in simple terms before a subject is enrolled in the

study. The informed consent document contains a statement that the consent is freely given, that the subject is aware of the risks and benefits of entering the study, and that the subject is free to withdraw from the study at any time. Written consent will be given by the parent/guardian, after receiving detailed information on the study. Written informed consent must be obtained for each subject prior to enrolment in the study. The informed consent form will be signed and personally dated by the child's parent or guardian. The person who conducts the informed consent discussion should also sign and date the inform consent form. A copy of original signed informed consent form will be retained in the participant's chart and another will be provided to him. A participant who is unable to read or write will place an imprint of his finger in the place of a signature; in addition, an independent witness will sign the consent form to attest that the information in the consent form was orally conveyed to the participant. A copy of the inform consent document in English is provided in Annex 4.

i. Minors or subjects with cognitive impairment

Minor subjects (under 5 years of age) with or without cognitive impairment will be enrolled into this study after inform consent granted by the parent or guardian.

E. Subject Confidentiality

The investigator will ensure that the subject's anonymity is maintained. Participants will not be identified in any publicly released reports of this study. All records will be kept confidential to the extent provided by laws and regulations. The study monitors and other authorized representatives of the regulatory authorities may inspect all documents and records required to be maintained by the Investigator. All laboratory specimens, evaluation forms, reports, and other records that leave the site will be identified only by a coded number in order to maintain subject confidentiality.

F. Study Discontinuation

Implementation of SMC has already started the district of Koutiala, in Mali. The plan is to expand it to additional districts each year. It is expected that by the end of this project in 2017, SMC implementation will be expended to the whole country including the district of Ouelessebougou. PMI is one of key partners in Mali that is now supporting SMC implementation

in Mali. Should the extension of the SMC implementation delayed, the investigators will work with the National Malaria Control Program and PMI to insure that implementation of the SMC using the optimal approach in Ouelessebougou continue.

6. Waste Management and Monitoring Plan

MRTC has been conducting clinical research with NIAID/NIH for more than 20 years. As results of this collaboration the MRTC is equipped with CAP certified clinical laboratory. The staff is well aware and trained on the waste management and monitoring. Waste management and monitoring will performed according the SOP provided in Annex 2. The primary waste associated with the study includes biohazard waste (solid and liquid). Clinical and laboratory staff are responsible for safe disposable of biohazard waste according to the SOP. This responsibility includes segregating non-hazardous waste (like paper) from hazardous waste to minimize environment impact. Laboratory and clinics are supplied with protective clothing such as lab coat and disposable gloves and material to collect biohazard waste. IMEP program facility located at Ouelessebougou is equipped with an incinerator for the treatment of solid waste. Waste produced by outlined clinics will be treated locally if incinerator is available or will be brought to the central lab facility at Ouelessebougou. Sites equipped with incinerator will serve as the final disposal sites. A plan to vaccinate team members with hepatitis B vaccine will be implemented as part of the overall safety management prior to the study initiation. As with all clinical, laboratory and data procedure, SOP adherence is monitored on a regular basis by the program PIs.

7. Vertebrate Animal Welfare

Not applicable.

8. Capacity Development

The project will build capacity in the local health system and at MRTC. Staff of the health center in selected villages and at the district levels will be trained to carry out the SMC interventions with support of the project team composed of MRTC, LMIV and the National Malaria Control Program. Implementations, monitoring and evaluations tools will be developed and could be used in other districts. Cases of malaria will be properly diagnosed, treated and reported. Integrated diseases surveillance system will be strengthened. Junior scientists at MRTC will

work on this project as part of the continued training and capacity building at MRTC. The project will contribute to acquire new clinical lab and IT equipment and to maintain existing equipment that will be used for the project at MRTC. The data collected will be used as part of Master, MD, PharmD and PhD thesis at the University of Bamako and elsewhere. .

9. Data Dissemination Plan and Knowledge Transfer

The study findings will be presented and discussed during the regular meetings of the project management board (PB) including a representative of the National Malaria Control Program , PMI and CDC that will be established for this project. Each year the results of the study will be presented to the communities where the trials have been conducted and to the relevant local and national health authorities through ad-hoc meetings, data dissemination workshops and/or during the celebration of African Malaria Day in Mali. Results of the study will also be presented at national and international scientific meetings including malaria controls meetings and publications in peer reviewed journals. As with previous SMC trials the detailed reports and peer review publications will be submitted to the Global Malaria Program of the World Health Organization. Results will also be shared regularly with the local representations of the WHO and UNICEF as well as other partners in Mali through regular "health cluster" meetings, which take place every Thursday at the WHO office in Bamako and involve representatives of the Ministry of Health, the WHO, UNICEF, USAID and other health partners.

10. Detailed Timeline

Timelines of the key study activities are summarized in Table 1.

Table 1. Study main activities and timelines

	Months																																			
**Activities**	1*	2	3	4	5	6	7	8	9	10	11	12	13	14	15	16	17	18	19	20	21	22	23	24	25	26	27	28	29	30	31	32	33	34	35	36
Finalisation of the protocol, SOPs, CRFs	■	■																																		
Protocol Submission to EC		■	■																																	
Selection of the sub-districts and randomization				■																																
Training of the study staffs				■	■																															
Census				■	■																															
Cross-sectional survey (baseline beginning of the season)					■	■																														
SMC administration						■	■	■										■	■	■	■									■	■	■	■			
Passive surveillance						■	■	■	■	■	■	■	■	■	■	■	■	■	■	■	■	■	■	■	■	■	■	■	■	■	■	■	■	■		
End of season cross-sectional survey									■	■											■	■											■	■		
Database development & testing				■	■	■																														
Data management							■	■	■	■	■	■	■	■	■	■	■	■	■	■	■	■	■	■	■	■	■	■	■	■	■	■	■	■		
Data analysis & reports											■	■											■	■											■	■

* march 2014

11. Detailed Budget

Provide a detailed budget for the study and a detailed justification/narrative of the budget. Identify all sources of funding.

12. Annexes

- References/Citations
- Data collection forms or other instruments used
- Informed consent forms

Ministère de l'Enseignement Supérieur
et de la Recherche Scientifique

République du Mali

Un Peuple – Un But – Une Foi

UNIVERSITE DES SCIENCES TECHNIQUES ET TECHNOLOGIES DE BAMAKO

FACULTE DE PHARMACIE

Malaria Research & Training Center

<u>Titre</u> :

Optimisation de la mise en œuvre de la Chimioprévention du Paludisme Saisonnier (CPS) et ses effets sur l'acquisition de l'immunité anti palustre.

Version 1.1 du 16 Juin 2014

<u>Financé par:</u>

<u>USAID-NIH PEER Health</u>

Composition de l'Equipe

Principal Investigateur: Alassane Dicko, MD, DrPH

Malaria Research and Training Center (MRTC)
Faculté de Pharmacie, Université de Sciences Techniques et Technologies de Bamako
B.P. 1805; Bamako, Mali
Tel: 2023-2022 7440
Email: adicko@icermali.org

Co- Principaux Investigateurs: Patrick E. Duffy, MD
Michal Fried PhD

Laboratoire d'Immunologie et de Vaccinologie du Paludisme NIAID/NIH
Twinbrook 1, Room 1111
5640 Fishers Lane
Rockville, Maryland 20852 USA
Tel: 301 435-2177 Fax: 301 480-1958

Co-Investigateurs Seniors : Ogobara Doumbo, MD, PhD
Issaka Sagara, MD, MSPH

Malaria Research and Training Center
Faculté de Médecine et d'Odonto-Stomatologie et Faculté de Pharmacie
Université de Sciences Techniques et Technologies de Bamako

Diakalia Kone, MD, MSc. Ph
Mohamed Keita, MD
Programme National de Lutte contre le Paludisme;
Bamako; Mali

Co- Investigateurs Moctar Tounkara, MD, MPH
Amadou Barry, MD, MS
Youssoufa Sidibe, MD
Djibrilla Issiaka, MD
Halimatou Diawara, MD, MPH
Almahmoudou Mahamar, PharmD
Oumar Attaher, PharmD

Fanta Koita, PharmD Candidate

Malaria Research and Training Center
Faculté de Médecine et d'Odonto-Stomatologie et Faculté de Pharmacie
Université de Sciences Techniques et Technologies de Bamako

Statisticiens: Erin Gabriel, Ph.D.
Michael P. Fay, Ph.D.

NIAID, NIH
6700-B Rockledge Drive, MSC 7609
Bethesda, Maryland 20892-7609 USA

Date de début programmée: Juin 2014

Date de fin programmée: Mai 2017

ABREVIATIONS

AMA1: Antigène de la membrane apicale

AQ : Amodiaquine

ASC: Agent de santé communautaire

BPC: Bonnes pratiques cliniques

BPL: Bonnes pratiques de laboratoire

CAP: Collège des pathologistes américains

CDC: Centre pour le contrôle de la maladie et Prevention

CGP: Comité de gestion du projet

CPS: Chimioprévention du paludisme saisonnier

CSP: Circum sporozoite proteine

CTA: Combinaison thérapeutique à base d'artémisinine

Dhfr : Dihydrofolate réductase

DHPS: Dihydropteroate synthétase

DPF: Distribution à point fixe

DPP: Distribution porte à porte

EI: Evénement indésirable

EIG: Evénement indésirable grave

GE/FM: Goutte épaisse/Frottis mince

Hb: Taux d'hémoglobine

IMEP: Immuno-épidémiologie

LMIV: Laboratoire d'immunologie et vaccinologie du paludisme

MII: Moustiquaires impregnées d'insecticides

MRTC: Centre de recherche et de formation sur le paludisme

MSP1: Merozoite de surface proteine 1

NIH: Instituts Nationaux de Santé des Etats Unis d'Amérique

OMD: Objectifs du millénaire pour le développement

OMS: Organisation mondiale de la Santé

PCR: Polymerase chaine réaction

Pfcrt: Plasmodium falciparum chloroquine résistance transporter

pfmdr1: Plasmodium falciparum résistant à de multiple médicaments

PID: Pulvérisation intra domiciliaire

PMI: Initiative du président américain pour la lutte contre le paludisme

PNLP: Programme national de lutte contre le paludisme

POS: Procédures opératoires standardisées

SP: Sulfadoxine - pyrimethamine

TDR: Test de diagnostic rapide

UNICEF: Fonds des nations unies pour l'enfance

USAID: Agence internationale des Etats Unis pour le développement .

Table des matières

1. Résumé……………………………………………………………………………..9

2. Introduction 11

2.1. Utilisation potentielle des résultats 13

3. Objectifs et critères de jugement 14

3.1. Objectifs de l'étude 14

3.1.1. Objectif général 14

3.1.2. Objectifs spécifiques...14

3.2. Critères de jugement de l'étude 15

4. schéma de l'étude 15

4.1 Lieu d'étude……………………………………………………………………...15

4.2 Schéma général...16

4.3 Description de la population d'étude..18

4.4 Recensement et randomisation 18

4.5 Critères d'éligibilité 18

4.6 Interventions 19

4.7 Suivi et mesure des critères de jugement..20

4.8 Définitions 21

4.9 Procédures de laboratoire 21

4.10 Arrêt de l'étude..24

4.11 Les critères de retrait des sujets et leur gestion...24

4.12 Assurance qualité / contrôle de qualité des données et de la collecte.................................24

4.13 Plans de gestion des catastrophes...24

5. Considérations statistiques et analyse des données..25

5.1 Taille de l'échantillon et calcul de la puissance..25

5.1.2 Surveillance passive. 25

5.1.3 Paramètres immunologiques. 25

5.2 Analyses intermédiaires prévues..26

5.3 Plan d'analyse final : 26

6. Considérations éthiques et protection des sujets humains...27

6.1 Norme éthique 27

6.2 Comité d'éthique...**Error! Bookmark not defined.**

6.3 Processus de consentement....27

6.3.1 Permission communautaire..27

6.3.2 Consentement individuel..28

6.4 Risques potentiels...**Error! Bookmark not defined.**8

6.5 Evènéments indésirables..29

6.6 Avantages potentiels...31

6.7 Confidentialité 31

6.8 Mineurs ou sujets souffrant de troubles mentaux. ....**Error! Bookmark not defined.**

7. Fin de l'étude..32

8. Plan de monitorage et de gestion des déchets 32

9. Développement des capacités..33

10. Plan de publication des données et le transfert des connaissances 33

11. Chronologie detaillée...33

1. Résumé

Basée en partie sur les études de base que nous avons menées au Mali, la CPS a été approuvée en Mars 2012 par l'OMS comme une politique de lutte contre le paludisme dans les pays où la transmission du paludisme est saisonnière tel que le Mali. La stratégie est efficace et à faible coût pour réduire la mortalité chez les enfants dans ces zones. Malgré l'énorme avantage de la CPS sur l'infection et la maladie palustre, l'approche optimale pour la distribution de la CPS reste à déterminer et il n'existe pas de données sur l'effet à long terme de cette stratégie sur l'acquisition de l'immunité anti palustre. Bien que la distribution à un point fixe (DPF) combinée avec un traitement non directement supervisé (TNDS) par les agents de santé communautaires soit intéressante pour la mise en œuvre de la CPS, il est nécessaire de l'évaluer et de comparer à d'autres modes de distribution. Les objectifs consistent à identifier la méthode la plus efficace pour distribuer la CPS, et obtenir des informations sur l'impact à long terme de la CPS sur l'immunité anti palustre. Plus précisément, i) déterminer le mode optimal et la fréquence optimale de la distribution de la CPS ; ii) comparer des mesures quantitatives de l'immunité chez les enfants qui ont reçu la CPS et ceux qui n'ont pas reçu la CPS sur une période de trois ans.

Le schéma est un essai randomisé par grappes sur trois ans. Les populations cibles sont les enfants âgés de 3-59 mois vivant dans les villages des aires de sante sélectionnées de Oueléssébougou, Mali. En première année, les villages de quatre aires de santé seront randomisés en quatre groupes (DPF + TDS ; DPF + TNDS ; DPP + TDS ; DPP + TNDS). Le mode optimal de distribution sera choisi en fonction de la couverture CPS au cours de la première année, et sera ensuite mis en œuvre dans les villages de deux autres aires de santé. Les villages de ces deux aires de santé seront randomisés en deux groupes. Les enfants du premier groupe recevront trois passages de CPS et ceux du deuxième groupe recevront quatre passages de CPS pour déterminer la fréquence optimale de la CPS sur la base du taux d'incidence du paludisme clinique mesuré par la surveillance passive. Les enfants dans les quatre aires de santé sélectionnés au cours la première année continueront de recevoir trois passages de CPS la 2ème année en utilisant le mode optimal de distribution. Au cours de la 3ème année, les enfants dans les aires de santé choisies au hasard recevront la CPS par le système de distribution optimale déterminée au cours de la première et la deuxième année. Les réponses immunitaires seront mesurées et comparées entre la cohorte d'enfants recevant la CPS et la cohorte d'enfants ne recevant pas de CPS, pour évaluer l'impact de la CPS sur les réponses d'anticorps antipaludiques clés au cours de la période des trois ans à l'aide d'enquêtes transversales au début et à la fin de la saison de transmission. La prévalence de l'infection palustre, l'anémie et des marqueurs moléculaires de la résistance de *P. falciparum* à la SP et AQ seront mesurés au cours de ces enquêtes transversales comme critères d'évaluation secondaires sur un sous-échantillon d'enfants sélectionnés au hasard.

Le résultat attendu de l'étude sera la détermination d'une approche optimale qualifiée pour la distribution de la CPS au Mali, et une compréhension élargie de son impact sur la santé et l'immunité anti palustre.

2. Introduction

Plus de 200 millions de personnes vivent dans des zones où la transmission du paludisme est très saisonnière. Le paludisme demeure dans le monde la cause majeure de morbidité et de mortalité dans le monde, on estime entre 610 000 à 971 000 cas de décès dus au paludisme chaque année. L'Afrique sub-saharienne est touchée de façon disproportionnée, souffrant de 91% des décès dus au paludisme avec 88 % survenant chez les enfants de moins de 5 ans [1]. En l'absence d'un vaccin, des stratégies de contrôle simples et efficaces sont nécessaires d'urgence pour réduire le fardeau du paludisme en Afrique sub-saharienne. La lutte anti vectorielle, en utilisant des moustiquaires imprégnées d' insecticide (MII), des rideaux imprégnés d'insecticide, ou la pulvérisation intra domiciliaire (PID) peut réduire significativement la mortalité et la morbidité du paludisme [2], mais dans les zones de forte transmission, ces interventions procurent une protection partielle et des mesures de contrôle supplémentaires sont nécessaires.

Il a été récemment démontré que la Chimio prévention du Paludisme Saisonnier (CPS) précédemment connue sous le nom de traitement préventif intermittent du paludisme chez les enfants, réduit l'infection palustre ainsi que le paludisme maladie de plus de 80% chez les enfants maliens, encourageant l'OMS à approuver la CPS comme politique pour les pays sahéliens en Mars 2012 [3,4]. La CPS consiste à l'administration du traitement curatif complet de médicament antipaludique au cours de la saison de haute transmission du paludisme pour prévenir le paludisme avec comme objectif de maintenir les concentrations de médicaments antipaludiques à dose thérapeutique dans le sang tout au long de la période de haute transmission du paludisme. La recommandation de l'OMS indique qu'un traitement curatif complet de la sulfadoxine - pyriméthamine + amodiaquine (SP + AQ) doit être donné aux enfants âgés de 3 à 59 mois à un mois d'intervalle en commençant au début de la saison de transmission du paludisme avec un maximum de quatre rounds (passages) au cours de la saison de transmission du paludisme [4].

Il a été prouvé que la stratégie est très efficace, sûre et rentable pour la prévention du paludisme chez les enfants de moins de 5 ans dans les zones à forte transmission saisonnière du paludisme, comme la région du Sahel en Afrique [4].

Bien que plusieurs approches possibles puissent être utilisées pour mettre en œuvre la stratégie, les données sont insuffisantes pour recommander une méthode d'administration standard de la CPS [5]. Deux études ont comparé la distribution de la CPS par les agents de santé communautaires et les établissements de santé au Ghana et en Gambie [6, 7]. En Gambie une couverture plus élevée de la CPS a été obtenue quand elle est assurée par les agents de santé communautaire (ASC) que par le personnel local des centres de santé [7] tandis qu'au Ghana, la couverture de la CPS était similaire lorsqu'elle a été distribuée par les ASC

ou par le personnel des établissements de santé (distribution par la communauté 69% et distribution par les établissements de santé 66%) [6]. Aucune étude n'a comparé le bénéfice du traitement complet directement supervisé par rapport au traitement non directement supervisé ou la distribution porte à porte à la distribution point fixe lorsque la stratégie est délivrée par les agents de santé communautaires.

Les principales études d'efficacité au Mali, au Sénégal et au Burkina Faso qui ont conduit à l'évaluation et à la recommandation de la stratégie ont évalué l'efficacité de deux ou trois passages de traitement de la CPS, et il n'y a pas de données concernant le bénéfice additionnel d'un 4ème passage de traitement de CPS dans la recommandation de l'OMS. Dans cette étude, nous nous proposons d'évaluer l'efficacité du 4 ^{eme} passage du traitement CPS contre les épisodes d'accès palustre au Mali

Nos études antérieures ont démontré que la CPS réduit l'infection palustre et le paludisme maladie de plus de 80% chez les enfants au Mali. En plus de l'effet protecteur du traitement intermittent, d'autres facteurs tels que la méthode de distribution du traitement ont contribué à la réussite de ces essais. La politique de l'OMS pour la mise en œuvre de la CPS est basée sur des essais menés au Mali et dans d''autres pays. Cependant des facteurs essentiels tels que la méthode optimale de distribution pour la mise en œuvre au niveau des pays et permettant d'atteindre un taux de réussite similaire à celui observé dans les essais cliniques, reste à déterminer.

Cette étude est conçue pour répondre à l'objectif principal urgent, qui est d'identifier les méthodes les plus efficaces et rentables pour la distribution de la CPS au Mali, en vue de soutenir la mise en œuvre de la CPS au Mali. Une distribution optimale de la stratégie sera d'un apport significatif à la réalisation des objectifs de l'Initiative du Président Américain pour la lutte contre le paludisme (PMI) et les Objectifs du Millénaire pour le Développement (OMD). La méthode la plus efficace de la distribution sera évaluée en comparant: la distribution porte -à-porte (DPP) à de la distribution point fixe (DPF) et le traitement directement supervisé (TDS) au traitement non directement supervisé (TNDS).

Un autre objectif spécifique est de déterminer le nombre de rounds de traitement. Efficace dans les essais qui ont servi de base pour l'élaboration de la politique de CPS par l'OMS, les enfants ont reçu trois rounds de traitement par an, la politique de l'OMS suggère jusqu'à quatre rounds de traitement par an. En tenant compte de la logistique associée à chaque round de traitement, il est essentiel de déterminer si un 4ème round (passage) aura un avantage supplémentaire par rapport à trois rounds (passages) de traitement.

Malgré l'énorme avantage fourni par la CPS, l'une des préoccupations est qu'elle peut compromettre l'acquisition de réponses immunitaires protectrices, ce qui augmente les risques de la maladie dans les années ultérieures. Des études menées au Mali et dans d'autres pays ont comparé la sensibilité au paludisme chez les enfants qui ont reçu l'intervention sur une seule saison et le groupe de contrôle pendant la saison de

transmission suivante du paludisme et ont trouvé une petite augmentation du paludisme clinique chez les enfants qui ont reçu l'intervention [3 ;8 ;9 ;13]. Aucune de ces études d'efficacité n'a évalué l'impact de la CPS sur l'immunité au paludisme lorsqu'il est administré pendant plus d'une saison de transmission. Dans les conditions d'intervention à long terme, nous faisons l'hypothèse que l'immunité contre les antigènes du paludisme au stade hépatique (reflétant l'exposition aux piqûres de moustiques infectés) sera similaire chez les enfants recevant la CPS à celle des enfants qui ne recevront pas la CPS, mais l'immunité aux antigènes de stade sanguin sera nettement diminuée (reflétant la protection contre l'infection au stade sanguin). Dans ce protocole, les enfants recevront l'intervention de manière séquencée et seront suivis pendant une période de 3 ans. Nous étudions actuellement l'acquisition de l'immunité au paludisme chez les enfants recrutés à la naissance dans le cadre d'une étude immuno- épidémiologique (IMEP) de cohorte à Ouélésségbougou, où nous prévoyons de recruter jusqu'à 2000 enfants qui seront suivis pendant 5 ans. Nous en tirerons partie en utilisant une partie de cette cohorte d'enfants en tant que groupe de contrôle pour l'étude de l'intervention proposée. Cela donnera un aperçu de l'acquisition d'une immunité contre le paludisme et pourra indiquer si l'arrêt de la CPS de longue durée augmentera ultérieurement le risque de paludisme et si des mesures de contrôle supplémentaires seront nécessaires.

Dans notre précédente étude qui a évalué l'impact de la CPS au cours d'une saison sur la résistance à la SP, il n'y avait pas de différence dans la fréquence des marqueurs de résistance de SP entre les parasites recueillis dans les groupes qui ont reçu le traitement et les groupes qui non pas reçu le traitement. On ne sait pas si la mise en œuvre de la CPS à long terme ne va pas accélérer l'accumulation de parasites résistants, ou le développement de la résistance à SP et AQ. L'évaluation du TPI pendant la grossesse en Tanzanie, une zone avec une résistance généralisée à la SP, a montré que le TPI pendant la grossesse était associé à une augmentation de la densité parasitaire placentaire et l'inflammation. L'étude actuelle va permettre la comparaison des fréquences des marqueurs de résistance à SP et à AQ au début de l'étude et à chaque année de suivi. Le succès de la CPS en Afrique de l'Ouest est dû à une propagation limitée de parasites résistants aux médicaments, cependant, les changements dans la population de parasites doivent être surveillés pour s'assurer de l'efficacité de cette stratégie.

2.1. Utilisation potentielle des résultats

La CPS est une nouvelle intervention approuvée par l'OMS comme politique en Mars 2012. Les principaux objectifs ainsi que les objectifs les plus urgents de ce projet sont d'identifier la méthode la plus efficace pour la distribution de la CPS et d'obtenir plus d'informations sur l'impact à long terme de la CPS sur l'immunité contre le paludisme. Le Mali est un des pays soutenus par l'Initiative du président américain

contre le paludisme (PMI) mis en œuvre par l'USAID. Le but de PMI est de réduire la mortalité liée au paludisme de 50% dans certains pays par la réalisation de 85% de couverture des interventions préventives et curatives approuvées, en appuyant le Programme national de lutte contre le paludisme. Trouver la méthode optimale de distribution de la CPS permettra un déploiement idéal de la stratégie par pays pour un impact plus élevé sur la mortalité y compris la mortalité du paludisme au Mali et contribuer à la réalisation pour atteindre l'objectif de développement du millénaire et les objectifs du PMI. Le projet permettra également d'évaluer l'effet de la CPS sur l'immunité contre le paludisme lorsqu'il est administré pendant trois ans consécutifs de sorte que plus de compréhension sur l'immunité contre le paludisme sera obtenue et des mesures appropriées pourront être prises pour accompagner la mise en œuvre de la stratégie au besoin. Le projet sera réalisé par le système de santé local. Le personnel du centre de santé dans les villages sélectionnés sera formé pour effectuer les interventions CPS avec le soutien de l'équipe de projet composée de MRTC, LMIV et le Programme National de Lutte contre le Paludisme (PNLP). Le projet collaborera avec la mission PMI de l'USAID à Bamako.

3. Objectifs et critères de jugement

3.1. Objectifs de l'étude

3.1.1. Objectif général

L'objectif général est d'identifier la méthode la plus efficace pour la distribution de la CPS, et d'obtenir plus d'informations sur l'impact de la CPS à long terme sur l'immunité contre le paludisme.

3.1.2. Objectifs spécifiques

- Déterminer le mode optimal et la fréquence optimale d'administration de la CPS
- Comparer les mesures quantitatives de l'immunité chez les enfants qui ont reçu la CPS et les enfants qui n'ont pas reçu la CPS.
- Evaluer l'impact de la CPS à long terme sur la fréquence des marqueurs moléculaires de la résistance de *P. falciparum* à la SP et AQ.

3.2. Critères de jugement de l'étude

3.2.1 Les critères de jugement principaux sont les suivants:

- ***La couverture de la CPS*** pour la détermination du mode optimal de distribution lors de la première année. La couverture de la CPS sera définie comme la proportion d'enfants ayant reçu les trois doses de traitements à chacun des trois passages de la CPS.
- L'incidence des accès palustres pour la fréquence optimale de la CPS au cours de la 2ème année. L'accès palustre sera défini comme les signes et des symptômes évocateurs de paludisme avec TDR positif et / ou une goutte épaisse ou frottis sanguin positif aux formes asexuées de parasites.

3.2.2. Les critères de jugement secondaires sont les suivants:

- La prévalence de l'infection palustre définie comme la proportion des enfants ayant une goutte épaisse ou frottis sanguin positif et la densité parasitaire mesurée en An 1, 2 et 3.
- La prévalence de l'anémie modérée définie comme la concentration d'hémoglobine < 8 g / dl mesurée par un hémoglobinomètre.
- Les réponses immunitaires cellulaires et humorales antipaludéennes aux parasites du paludisme.
- Les niveaux de cytokines et d'autres réponses de l'hôte mesurés dans le sérum / plasma ou cellules immunitaires.
- La fréquence des mutations au niveau des codons 51 , 59 et 108 du gène de la dhfr 437 et 540 du gène de la DHPS, codon 76 dans la *P.falciparum* gène transporteur de la chloroquine (pfcrt), et le codon 86 du gènes de résistance à des médicaments multiples *P. falciparum* (pfmdr1)

4. Schéma de l'étude

4.1 Lieu d'étude :

L'étude aura lieu dans le district sanitaire de Ouéléssébougou. Le district sanitaire de Ouéléssébougou couvre 14 aires de santé avec une population totale estimée à 237000 habitants en 2014. La commune de Ouéléssébougou est situé dans le cercle de Kati, région de Koulikoro et Bamako au Mali. Ouelessebougou est le chef lieu de sous-préfecture et de la commune du même nom situé à 80 km au sud de Bamako sur la route Bamako-Sikasso. Ouelessebougou se trouve dans la zone de transmission saisonnière longue de quatre à six mois. En 2008, dans cette zone le taux d'incidence du paludisme chez les moins de cinq ans était d'environ deux épisodes/enfant/an et le taux d'incidence du paludisme grave selon les critères de l'OMS était d'environ 1-2% dans cette tranche d'âge pendant la saison de transmission.

4.2 Schéma général

L'étude utilisera un schéma randomisé par grappe sur trois ans. Huit aires de santé seront choisies au hasard parmi les 13 aires de santé de Ouéléssébougou mais à l'exclusion de l'aire de santé où les inclusions et le suivi des enfants de cohorte l'étude IMEP sont en cours. Les huit aires de santé seront randomisées afin que l'intervention commence dans quatre aires de santé au cours de la première année (An 1), dans deux aires de santé au cours de la deuxième année (An 2) et les deux autres aires de santé restantes la troisième année (An 3).

La population cible sera les enfants âgés de 3-59 mois résidents des villages sélectionnés dans le district de Ouéléssébougou, région de Koulikoro, Mali.

La première année, la mise en œuvre de la CPS aura lieu dans quatre aires de santé choisies au hasard à Ouéléssébougou. Les villages de ces aires de santé seront randomisés selon un ratio 1:1 pour recevoir soit par distribution porte à porte (DPP) ou par distribution point fixe (DPF) de CPS. Ils seront également randomisés pour recevoir la CPS sous forme de traitement directement supervisé (TDS) ou un traitement non directement supervisé (TNDS). Un recensement de la population cible (les enfants âgés de 3-59 mois) sera réalisée dans tous les villages sélectionnés avant l'intervention. Après le recensement, des échantillons de sang seront prélevés dans un sous-ensemble aléatoire de la population de l'étude afin de recueillir des données de base sur les réponses cellulaire et humorale contre les antigènes du paludisme, la prévalence parasitaire et la densité sur goutte épaisse /frottis sanguins ainsi que la fréquence des marqueurs moléculaires de la résistance à la SP et AQ au début de la saison de transmission. La liste de recensement sera utilisée pour réaliser l'intervention et sera régulièrement mis à jour. La CPS consiste l'administration de traitement complet de la sulfadoxine-pyriméthamine + amodiaquine (SP + AQ) à intervalle mensuel pendant la saison de transmission du paludisme à partir d'Août 2014. Les enfants seront suivis passivement pour la détermination de l'incidence du paludisme maladie (accès palustres simples et graves). Une enquête transversale sera réalisée après le troisième round en Novembre / Décembre pour déterminer la couverture de la CPS, la prévalence de la parasitémie et de l'anémie ainsi que des réponses immunitaires humorales et cellulaires chez un certain nombre d'enfants choisis au hasard. Les données recueillies au cours de la 1ère année seront analysées afin de déterminer la méthode de distribution optimale (DPP + TDS ou DPP + TNDS ou DPF + TDS ou DPF + TNDS) basée principalement sur la couverture de la CPS et secondairement sur l'incidence des accès palustres. La différence dans la couverture de la CPS devrait se refléter sur l'incidence du paludisme maladie, et celui-ci sera utilisé pour appuyer les données sur la couverture de la CPS. S'il n'y a pas de différence statistiquement significative entre les bras en termes de

couverture de la CPS et l'incidence de la maladie, la méthode la plus simple de distribution sera recommandée pour la 2ème année.

La 2ème année, la CPS sera mise en œuvre en utilisant le système optimal de distribution (déterminé en An 1) dans deux aires de santé choisies pour la 2eme année après un recensement des enfants de moins de 5 ans. Les villages de ces deux aires de santé nouvellement sélectionnées seront randomisés pour recevoir soit trois, soit quatre rounds de CPS pendant la saison de transmission à partir d'Août. Les données de base sur l'infection palustre, les marqueurs de résistance à la SP et AQ et les réponses immunitaires aux antigènes du paludisme seront recueillies à l'inclusion avant l'intervention dans ces deux aires de santé à travers une enquête transversale. Afin d'évaluer l'effet du quatrième round de la CPS, les enfants seront suivis passivement pour mesurer le taux d'incidence du paludisme maladie et une enquête transversale sera effectuée à la fin de la saison de transmission du paludisme en Novembre / Décembre sur un échantillon d'enfants sélectionnés au hasard pour déterminer la prévalence de l'infection du paludisme, l'anémie, et les réponses immunitaires humorales et cellulaires. Les enfants qui ont participé à la 1ère année continueront de recevoir trois rounds de CPS la 2ème année en utilisant la meilleure méthode de distribution identifiée au cours de la 1ère année. Le choix du nombre optimal de doses sera basé sur l'incidence des accès palustres entre les deux bras d'enfants dans les deux aires de santé nouvellement sélectionnées. Une différence absolue de 7,5 épisodes par 100 enfants par an sera considérée comme significative.
Au cours de la 3ème année, tous les enfants de l'étude dans les huit aires de santé sélectionnées recevront la CPS par la méthode et la fréquence optimale déterminées la 1ère et la 2ème année et seront suivis passivement pour les épisodes de paludisme cliniques jusqu'à la fin de la saison de transmission du paludisme. Les échantillons de sang veineux seront recueillis au début (Juillet / Août) et à la fin de la saison de transmission (Novembre/Décembre) sur un sous-ensemble d' enfants choisis au hasard pour évaluer les réponses humorales et cellulaires à des antigènes du paludisme ainsi que la prévalence de l' infection palustre chez les enfants qui ont reçu la CPS pendant une, deux et trois saisons de transmission du paludisme. Ces résultats seront également comparés à ceux des enfants de la cohorte IMEP qui n'ont jamais reçu la CPS, dans une analyse séparée, en tenant compte des différences et des similitudes entre les cohortes.

4.3 Description de la population d'étude

La population de l'étude se composera des enfants âgés de 3-59 mois vivant dans les villages des aires de santé concernées du district sanitaire de Ouéléssébougou. L'étude comprendra jusqu'à huit aires de santé du district de Ouéléssébougou avec une taille pouvant aller jusqu'à 10 800 enfants de moins de cinq ans.

4.4 Recensement et randomisation

Une fois la permission communautaire est obtenue, un recensement des enfants de moins de 5 ans sera entrepris et tous les ménages avec des enfants de moins de 5 ans seront identifiés. Au moment du recensement, un screening préliminaire des enfants potentiellement éligibles sera effectué. Les enfants potentiellement éligibles seront identifiés et les consentements éclairés seront obtenus avant administration de la CPS et la participation aux enquêtes transversales. Les enfants inscrits dans l'étude se verront attribuer un numéro d'identification unique et leurs données démographiques (date de naissance et/ou l'âge et le sexe) seront collectées. Le recensement sera mis à jour à chaque round de la CPS et les participants potentiellement éligibles (tels que les enfants qui n'ont pas atteint l'âge de trois mois au cours du précédent round) seront identifiés et screenés pour éligibilité et inclusion dans l'étude. La mise à jour du recensement permettra de recueillir des informations sur les migrations et les décès d'enfants inscrits à l'étude pour l'estimation précise du dénominateur sous forme de personne - temps de suivi.
L'unité de randomisation sera le village, et tous les enfants éligibles d'un village seront alloués au même bras de l'étude (DPP + TDS ou DPP + TNDS ou DPF + TDS ou DPF + TNDS) au cours de la 1ère année pour éviter toute confusion. Les villages dans les aires de santé sélectionnées seront randomisés selon un ratio 1:1 pour recevoir soit la distribution porte -à-porte (DPP) ou la distribution point fixe (DPF) de CPS. Ils seront également randomisés pour recevoir la CPS comme traitement directement supervisé (TDS) ou un traitement non directement supervisé (TNDS). Au cours de la 2ème année, les villages des deux districts nouvellement choisis au hasard seront randomisés en deux groupes pour recevoir soit trois ou quatre rounds (passages) de CPS.

4.5 Critères d'éligibilité

Les critères d'éligibilités seront ceux de l'Organisation Mondiale de la Santé. Les enfants des deux sexes âgés de 3-59 mois au début de chaque période d'administration du médicament seront éligibles à l'inclusion dans l'étude, si le consentement parental est obtenu. Les enfants de moins de trois mois à la date du début de l'administration de médicaments ne seront pas inclus, puisque la CPS n'est pas recommandée pour les enfants dans ce groupe d'âge dont le risque de contracter le paludisme est faible. Les enfants qui auront

atteint l'âge de 59 mois ou plus à la fin de la première ou de la deuxième année de traitement ne recevront pas de CPS dans les années ultérieures.

Les critères de non inclusion et d'exclusion seront limités autant que possible pour rendre les résultats de l'essai largement applicables et comprendront :

- maladie chronique grave,
- une allergie connue à l'un des médicaments de l'étude,
- les sujets avec une sérologie VIH positive connus, et sous prophylaxie au cotrimoxazole.

Les enfants atteints de paludisme clinique aigu au moment de l'administration du médicament de la CPS seront traités par CTA et ne recevront pas les médicaments de la CPS pendant ce round conformément à la recommandation de l'OMS. Ils recevront des médicaments de la CPS au cours des rounds ultérieurs.

4.6 Interventions

La CPS consiste à l'administration de traitement complet avec la sulfadoxine-pyriméthamine + l'amodiaquine (SP + AQ) à des intervalles mensuels pendant le pic de la saison de transmission du paludisme (d'Août - à Octobre ou Novembre selon l'année de l'étude et le bras de l'étude). Au cours de chaque round (passage), les enfants âgés de 3-11 mois recevront 75 mg de AQ administré une fois par jour pendant 3 jours et une dose unique de SP 250/12.5 mg, tandis que les enfants âgés de 12-59 mois recevront 150 mg d'AQ donnés une fois par jour pendant 3 jours et une dose unique de SP 500/25 mg. La dose unique de SP sera administrée seulement le premier jour, en même temps que la première dose d'AQ. Les enfants seront observés pendant 30 minutes après l'administration du médicament. Si des vomissements surviennent au cours de cette période, les médicaments seront ré- administrés. Si des vomissements se reproduisent ce sera noté, mais les médicaments ne seront pas donnés une troisième fois. Ces enfants ne seront pas exclus de l'essai et ils seront éligibles à recevoir les médicaments les rounds suivants.

Dans les villages choisis pour la distribution porte -à-porte (DPP), les agents de santé communautaires (ASC) se rendront dans chaque concession pour administrer les médicaments de la CPS. Dans les villages choisis pour la distribution point fixe (DPF), les enfants recevront la CPS à un point fixe du village. L'administration de ces trois jours de traitements à chaque round sera effectuée par l'ASC pour les enfants affectés au TDS, tandis que pour les enfants affectés TNDS, le traitement de la première journée sera donné par les ASCs mais les traitements des deuxième et troisième jours seront donnés par les parents/tuteurs à la maison. La CPS sera mise en œuvre à l'aide des directives de mise en œuvre sur le

terrain, standardisées / élaborées par l'OMS [4] adaptées à la situation au Mali. Les outils de suivi et évaluation de la CPS seront développés et intégrées avec le système de santé au Mali.

4.7 Suivi et mesure des critères de jugement

La surveillance de survenue de paludisme clinique commencera au moment de la première administration du médicament à l'étude en Août et se poursuivra jusqu'à la fin de la saison de transmission de la troisième année (Novembre/ Décembre 2016). Les épisodes d'accès palustre seront détectés à l'aide d'un système de surveillance passive. Les données seront recueillies par les agents de santé, y compris les agents de santé communautaires. Les épisodes cliniques de paludisme qui surviennent dans les centres de santé de l'étude ou dans les communautés de l'étude seront enregistrés tout au long de la période d'étude dans tous les villages de l'étude. Les enfants ayant une maladie évoquant le paludisme seront testés pour le paludisme avec le TDR et/ ou une goutte épaisse/ frottis de sang par le personnel du centre de santé et/ou les agents de santé communautaires et traités selon les recommandations du Programme National de Lutte contre le Paludisme. Les cas de paludisme clinique seront enregistrés sur un formulaire standardisé (cahier d'observation). La formation de recyclage du personnel sera effectuée chaque année pour assurer la standardisation dans l'enregistrement des symptômes et des signes qui sont utilisés pour faire un diagnostic et la classification des épisodes d'accès palustre.

Chaque année, au début et à la fin de la saison de transmission du paludisme, une enquête transversale sera réalisée dans un échantillon aléatoire d'enfants pour évaluer la prévalence de l'infection palustre, l'anémie et les paramètres immunologiques. La couverture de la CPS sera évaluée par enquête transversale à la fin de la saison de transmission à travers des interrogatoires des parents/tuteurs et en se référant aux informations sur les cartes d'administration de CPS. Les enfants seront examinés, et un échantillon de sang sera obtenu pour les analyses, y compris le dosage du taux d'hémoglobine, la préparation d'une goutte épaisse/frottis sanguin pour la microscopie et l'analyse des réponses immunitaires cellulaires et humorales aux antigènes connus (CSP, MSP1, AMA1) et les nouveaux antigènes de *P. falciparum* pendant les phases hépatiques et sanguines.

4.8 Définitions

Les définitions suivantes seront utilisées au cours de l'étude.

Paludisme maladie : La définition principale nécessite: i) la présence de la fièvre (température axillaire > 37,5°C) ou des antécédents de fièvre au cours des dernières 24 heures; ii) l'absence de toute autre cause évidente de la fièvre, ou antécédent de fièvre; iii) la présence de parasitémie asexuée *de Plasmodium* détectée à goutte épaisse/ frottis sanguin et/ou au TDR.

Une définition secondaire du paludisme sera i) la présence des signes ou des symptômes évocateurs de paludisme; ii) l'absence de toute autre cause évidente des signes et symptômes; iii) la présence de parasitémie asexuée de *Plasmodium* à la goutte épaisse/ frottis mince ou au TDR.

Le paludisme grave : Le paludisme grave sera défini selon les critères de l'OMS [10].

Anémie. L'anémie (légère à sévère) est définie comme la concentration d'Hb< 11g/dl, une anémie modérée à sévère comme une concentration d'Hb< 8g/dL et une anémie sévère comme une concentration d'Hb < 5 g/dl.

Marqueurs moléculaires de la résistance à la SP et AQ. Les mutations au niveau des codons 51, 59 et 108 dans le gène dhfr, 437 et 540 dans le gène dhps, des mutations au codon 76 de Pfcrt et le codon 86 de pfmdr1 seront suivies.

4.9 Procédures de laboratoire

La détection du paludisme. Le TDR sera utilisé pour le diagnostic initial du paludisme afin de conduire le traitement selon les directives du PNLP. La prévalence du parasite au cours des enquêtes transversales sera déterminée à l'aide de la goutte épaisse /frottis mince (GE/FM). Les gouttes épaisses/frottis minces réalisés seront lus ensuite par deux microscopistes. Les lames qui sont jugées discordantes soit pour la positivité, soit pour la densité parasitaire, seront lues par un troisième lecteur. Le résultat final sera la médiane des résultats des deux ou trois lectures.

Dosage du taux d'hémoglobine. La concentration d'hémoglobine sera mesurée par la méthode colorimétrique en utilisant un hémoglobinomètre (Hemocue AB, Angelholm, Suède).

Les marqueurs moléculaires de la résistance à la SP et AQ: Des échantillons de sang collectés sur papier filtre des enfants ayant une infection mono- spécifique à P. falciparum a la GE/FM seront analysés par nested PCR pour les mutations au niveau des codons 51, 59 et 108 du gène de la dhfr, 437 et 540 du gène de la dhps, 76 de gène Pfcrt, et 86 du gène pfmdr1, selon les méthodes déjà publiées [11 ; 12 ; 13]. Les cas d'infection mixte seront classés comme mutants.

Les essais immunologiques comprendront les réponses immunitaires humorales contre les antigènes de la phase sanguine et de la phase hépatique, les réponses cellulaires aux antigènes de la phase hépatique, les niveaux de cytokines et d'autres réponses de l'hôte (par exemple, érythropoïétine) mesurées dans le sérum / plasma ou dans les cellules immunitaires/ sous-ensembles de cellules immunitaires. Les critères principaux de jugement immunologique de cette étude seront les réponses humorales à l'antigène CSP pré-érythrocytaire qui est le mieux caractérisé, et les antigènes de la phase sanguine MSP1 et AMA1. Tous les trois antigènes sont des candidats vaccin contre le paludisme et le Laboratoire d'Immunologie et Vaccinologie du Paludisme (LMIV) a développé des essais hautement standardisés pour soutenir le développement préclinique et clinique de vaccins basés sur ces antigènes. Les réponses aux CSP reflètent l'exposition de l'hôte à des formes de parasites pré-érythrocytaires (sporozoïtes et les formes intra hépatiques), tandis que les réponses à AMA1 et MSP1 reflètent l'exposition à des formes de la phase sanguine du parasite.

En général les mécanismes immunitaires qui interviennent dans la protection acquise naturellement contre le paludisme ne sont pas définitivement établis. Pour cette raison, nous examinerons un certain nombre de réponses immunitaires humorales et cellulaires comme aspects secondaires et exploratoires de l'étude. Par exemple, les formes pré-érythrocytaires du parasite, lorsque atténuées, peuvent induire une immunité stérile chez les humains, et les données disponibles suggèrent que différentes réponses immunitaires cellulaires et humorales peuvent contribuer à la protection. Pour les critères immunologiques secondaires de cette étude, nous allons étudier les réponses immunitaires humorales et cellulaires à la CSP et de nouveaux antigènes pré - érythrocytaires qui peuvent avoir un rôle dans l'immunité protectrice. Le laboratoire LMIV a identifié plusieurs antigènes de *P. falciparum* dont les orthologues confèrent une

immunité protectrice pré-érythrocytaire dans les modèles de paludisme de la souris, y compris PFL1995c, LISP1 et SAP1.

Pour une immunité de la phase sanguine, il est connu que les anticorps IgG naturellement acquis peuvent éliminer les parasites et guérir les symptômes lorsqu'ils sont administrés comme thérapie passive, mais les cibles de ces anticorps sont inconnues. En plus de nos critères d'évaluation primaires de niveaux d'anticorps contre AMA1 et MSP1, nous allons également mesurer l'activité fonctionnelle des anticorps à la phase sanguine comme critères d'évaluation secondaires. Il s'agit notamment des anticorps qui bloquent la liaison des globules rouges parasités à l'endothélium et d'autres récepteurs, dont beaucoup de scientifiques croient qu'ils pourraient diminuer la séquestration des parasites et par conséquent la maladie palustre.
Sur la base des résultats de nos études immunologiques principales et secondaires, et les résultats cliniques de l'étude, nous pourrons procéder à des analyses exploratoires additionnelles. Par exemple, si les données cliniques ou de laboratoire suggèrent que la CPS module l'immunité protectrice; le LMIV a des outils de génomique, tels que les approches transcriptomiques ou protéomiques, pour élargir la surveillance des réponses de l'hôte (plasma, sérum, cellulaire) qui peuvent être impliqués dans l'infection et la maladie palustre.

4.10 Arrêt de l'étude

L'étude peut être interrompue s'il y a:

- La preuve de haut niveau de résistance de *P. falciparum* à la SP et AQ déterminé par la prévalence de dhps 540 mutation > 50 % ou de la résistance in vivo de P. falciparum à la SP+AQ >10 %
- Une réduction drastique de l'incidence du paludisme maladie en dessous de 0,1 épisode par enfant et par an sans intervention CPS.

4.11 Les critères de retrait des sujets et leur gestion

Les critères de retrait seront les suivants:

- Retrait du consentement éclairé
- Sujets VIH positif sous prophylaxie au Cotrimoxazole
- Allergie ou intolérance à un des médicaments de la CPS
- Voyage hors des villages de l'étude. Pour ces sujets, l'administration de médicaments de la CPS sera interrompue, mais les sujets continueront à recevoir des services de soins de santé de routine a travers les centres de santé de la zone.

4.12 Assurance Qualité / Contrôle Qualité des données et de la collecte

Un moniteur indépendant sera nommé par le promoteur (PEER ou NIAID/NIH) afin de veiller à ce que l'étude soit menée conformément au protocole de l'étude, au BPC, à l’éthique et aux exigences réglementaires et le respect des normes. Le moniteur pourra effectuer une visite de début, , une visite de fin de l’étude et au moins une visite supplémentaire chaque année. Le moniteur consultera de un sous-échantillon des données de laboratoire ainsi que des dossiers cliniques lors de chaque visite.

Des outils de mise en œuvre de la CPS seront élaborés en collaboration avec les autorités de santé du district. Les Procédures Opératoires Standardisées (POS) seront élaborées pour chacune des activités clés, y compris (le consentement, le recensement, l'administration de la CPS, l’examen clinique, le diagnostic et le traitement du paludisme, la gestion des échantillons ainsi que les techniques de laboratoire etc..). Les activités seront réalisées par un personnel compétent et formé. La formation initiale sur le protocole de l'étude, les BPC et BPL seront dispensées au personnel de santé dans les villages d'intervention ainsi que le personnel additionnel du projet. Une formation de recyclage sera effectuée au moins une fois par an. Les données seront recueillies dans les formulaires standards. Le contrôle de la qualité sera réalisé sur l'échantillon des TDR d'une manière régulière et les compte- rendus ainsi que les formations de recyclage seront effectués selon les procédures du centre de santé du district. Les médicaments de la CPS seront obtenus des sources fiables pour assurer la qualité. Les médicaments de la CPS, les CTA pour le traitement du paludisme simple, la quinine et l'artésunate injectables pour le traitement du paludisme grave ainsi que le TDRs pour le diagnostic du paludisme seront fournis par l'USAID PMI dans le cadre de leur soutien à la mise en œuvre de la CPS et à la lutte contre le paludisme au Mali. Le contrôle de qualité du médicament sera effectué par un laboratoire indépendant àtravers USAID - PMI et le CDC. Le diagnostic et le traitement du paludisme chez les enfants de moins de 5 ans est gratuit au Mali.

4.13 Plans de gestion des catastrophes

Les données de l'étude seront entrées régulièrement dans la base des données DataFax qui est soutenu et maintenu par le NIAID. Comme pour les données de l'étude IMEP en cours, l'équipe de gestion de DataFax sera chargée de maintenir les données au NIAID/NIH avec rigueur tout en assurant un système de soutien et de back-up. Le système a l'avantage de générer automatiquement les copies pdf des formes qui peuvent être imprimées pour remplacer les dossiers en cas de catastrophe.

5. Considérations statistiques et analyse des données

5.1 Taille de l'échantillon

Enquêtes transversales. Le calcul de la taille de l'échantillon pour les enquêtes transversales est basé sur la couverture de la CPS. En supposant une couverture moyenne de la CPS de 60% par la combinaison de méthodes non optimales (combinaison de deux groupes), un total de 300 enfants par bras sera nécessaire pour détecter 10% d'augmentation de la couverture moyenne des deux groupes mis en commun avec au moins 85% de la puissance en prenant en compte les tests multiples et l'utilisation de deux tests exacts de Fisher. Pour tenir compte de l'échantillonnage en grappe avec un minimum de 10 grappes par bras, une corrélation interclasse de 0,01 (sur la base d'une étude antérieure montrant la couverture vaccinale [15], et 5% de données manquantes, 446 enfants seront nécessaires par groupe, soit un total de 1784 enfants.

Surveillance passive En supposant un taux d' incidence du paludisme maladie de 0,3 épisodes par enfant et par an dans le bras qui recevra 3 rounds de CPS, pour détecter une différence absolue en taux d' incidence des accès palustres de 0,075 épisode par enfant par an (correspondant à une réduction de 25%) dans le bras de 4 rounds de CPS, avec un risque alpha unilatéral de 0,025 et une puissance de 80%, en utilisant le modèle quasi-poisson pour 20 grappes de taille égale par bras avec une corrélation interclasse de 0,01 et 5% des données manquantes, nous auront besoin de 1386 par bras , ou 2772 sujets au total.

Paramètres Immunologiques. Nous baserons nos calculs de la taille de l'échantillon pour les résultats immunologiques sur la réponse immunitaire humorale à AMA1. Nous choisissons cette réponse parce qu'AMA1 est hautement immunogenique parmi les antigènes du paludisme. En outre, cette intervention vise à la phase sanguine des parasites du paludisme pendant la saison de transmission, et la réponse à AMA1 reflète l'exposition à des parasites de la phase sanguine. Dans des études antérieures de sérologie chez des jeunes enfants qui participent à un essai de vaccin AMA-1 à Bancoumana, Mali [16], nous avons observé le pic du taux d'anticorps anti- AMA1 vers la fin de la saison de transmission du paludisme (chez les enfants recevant le vaccin de contrôle), avec des moyennes géométriques du niveau d'anticorps de 120 unités d'anticorps (IC à 95% de 70 à 205). Les niveaux d'anticorps juste avant le début de la saison de transmission du paludisme étaient de 40 (25-75) unités d'anticorps. Sur la base de ces données, 467 sujets par groupe fourniront 80% de puissance pour détecter une réduction de 2 fois ou plus du pic des niveaux d'anticorps anti-AMA1 chez les enfants recevant la CPS, en tenant compte de l'échantillonnage en grappes dans 10 grappes de taille égale avec une corrélation interclasse de 0,01 et 10% de données manquantes (échantillons peuvent être inadéquats ou se gâter lors de la collecte et du transport).

5.2 Analyses intermédiaires prévues:

A la fin de la première saison de transmission, les données seront analysées pour déterminer le mode optimal de distribution en comparant principalement la couverture de la CPS et secondairement le taux d'incidence des accès palustres entre les quatre groupes d'intervention. En outre, une analyse descriptive des fréquences de base des paramètres immunologiques et les marqueurs moléculaires de la résistance de *P. falciparum* à SP et AQ, sera également effectuée à la fin de l'An 1.

Les données recueillies la 2ème année seront utilisées pour déterminer la fréquence optimale des passages de la CPS en comparant principalement l'incidence des accès palustres et secondairement la prévalence de l'infection palustre à la fin de la saison entre les deux bras. L'analyse descriptive des paramètres immunologiques et des marqueurs de résistance aux médicaments sera également effectuée.

Les détails des méthodes statistiques sont fournis dans le plan de l'analyse finale ci-dessous.

5.3 Plan d'analyse final :

Les données seront saisies et vérifiées à l'aide de DataFax. La base des données nettoyée sera exportée vers Stata (Houston Texas USA) ou SAS pour l'analyse. Une analyse par intention de traiter sera utilisée, sauf pour les effets immunologiques pour lesquelles le nombre exact des traitements de la CPS reçus seront considérés.

La couverture de la CPS sera déterminée en utilisant les informations sur la carte CPS et de l'interview du parent ou tuteur et sera considérée comme étant la proportion d'enfants ayant reçu tous les traitements à tous les passages de la CPS pendant la saison de transmission. La proportion d'enfants ayant reçu au moins la première dose de traitement de la CPS sera également estimée comme critère secondaire de couverture de CPS. La couverture de la CPS sera comparée entre les bras avec les intervalles de confiance à 95% ajustés à l'échantillonnage en grappe. Le bras DPP + TNDS sera considéré comme groupe de contrôle.

Le recensement des enfants sera mis à jour à chaque passage et à la fin de la saison de transmission pour permettre une bonne estimation de personne - temps de suivi. Les cas de paludisme maladie seront enregistrés et le taux d'incidence des accès palustres sera défini comme le nombre d'épisodes d'accès palustres divisé par le nombre total des jours où l'enfant est à risque. Les enfants ne seront pas considérés comme étant à risque 21 jours après un épisode de d'accès palustre. Les taux d'incidence d'accès palustre seront comparés entre les bras de traitement en utilisant les modèles d'équation d'estimation généralisée pour estimer les ratios des taux d'incidence avec ajustement pour l'âge, les régimes précédents de la CPS et le manque d'indépendance entre les épisodes répétés chez le même enfant.

La proportion des autres critères secondaires de jugement tels que la prévalence l'infection palustre, la prévalence de l'anémie et la fréquence des marqueurs moléculaires de la résistance à SP et AQ et les effets indésirables associés aux médicaments de l'étude seront estimés et comparés entre les groupes à l'aide de modèles linéaires généralisés avec des intervalles de confiance à 95% , ajustés pour l'échantillonnage en grappes et les variables de confusion potentielles , tels que l'âge et l'utilisation des MII.

Les différences au niveau de référence seront comparées en utilisant le test de Chi -carré ou Fisher exact pour les variables qualitatives et par l'analyse de la variance pour les variables quantitatives qui sont normalement distribués. Les tests non paramétriques seront utilisés pour les variables qui n'ont pas une distribution normale.

6. Considérations éthiques et protection des sujets humains

6.1 Norme éthique

6.2 L'étude sera menée en conformité avec le protocole, les bonnes pratiques cliniques (BPC) et toutes les exigences réglementaires applicables. **Comité d'Éthique**

Le protocole et les formulaires de consentement et toute modification ultérieure seront soumis pour approbation au Comité d'éthique de la Faculté de Médecine de Pharmacie et d'Odonto-Stomatologie de l'Université des Sciences Techniques et Technologies de Bamako (US, DHHS/OHRP Federal Wide Assurance #: FWA00001769). Le consentement individuel oral sera obtenu avant l'administration de la CPS et un consentement individuel écrit sera obtenu pour la participation dans les enquêtes transversales.

6.3 Processus de consentement

6.3.1 Permission Communautaire

Les objectifs de l'étude et la méthode de réalisation de l'étude seront discutés avec le Ministère de la Santé et les autorités sanitaires du district avant le démarrage. L'approbation de la communauté sera recherchée à travers des réunions avec les dirigeants des localités de l'étude au niveau des aires de santé ainsi qu'au niveau des villages. Au cours de ces réunions, les procédures de l'étude (y compris des informations sur la mise en œuvre de la CPS) seront expliquées aux dirigeants. Le processus de permission communautaire pourra suivre les étapes suivantes :

- Explications et éclaircissements aux leaders, y compris le chef du village et les anciens sur les objectifs, les procédures ainsi que les risques pour les sujets de l'étude.
- Donner du temps aux leaders pour communiquer avec les membres de la communauté et nous informer sur toutes questions ou préoccupations de la communauté.
- Approfondir l'explication du protocole aux différents chefs de famille.

Il n'y aura pas de publicité spéciale pour une méthode particulière. Les parents seront informés et leur consentement sera demandé afin de faciliter la disponibilité des enfants du groupe d'âge cible à la maison dans les villages randomisés pour la distribution à domicile ou les conduire dans un lieu fixe qui sera déterminé dans les villages randomisés pour la distribution au point fixe. Les dirigeants auront l'occasion de poser des questions et donner leurs avis sur les meilleurs moyens d'encourager la participation de la communauté. Ils seront invités ainsi que les agents de santé communautaires à transmettre les informations aux membres des communautés.

6.3.2 Consentement individuel

Les documents du consentement éclairé seront utilisés pour expliquer les risques et les avantages de la participation à l'étude aux parents ou au tuteur en termes simples avant l'administration des médicaments de la CPS et les inclusions dans les enquêtes transversales. Un consentement éclairé oral sera demandé avant l'administration de la CPS pour chaque sujet. Un consentement écrit sera demandé aux parents / tuteurs des enfants sélectionnés pour les enquêtes transversales, après avoir reçu des informations détaillées sur l'étude. Le formulaire de consentement éclairé sera signé et daté personnellement par le parent ou le tuteur de l'enfant. La personne qui effectue la discussion sur le consentement éclairé doit également signer et dater le formulaire de consentement éclairé. Une copie du formulaire de consentement éclairé original signé sera conservé dans le dossier du participant et un autre sera fourni au parent / tuteur. Un participant qui est incapable de lire ou d'écrire posera une empreinte de son doigt à la place d'une signature, en outre, un témoin indépendant signera le formulaire de consentement pour attester que les informations contenues dans le formulaire de consentement ont été transmises oralement au parent / tuteur du participant.

6.4 Risques potentiels

Les risques pour les participants à l'étude sont mineurs. L'étude va utiliser uniquement les médicaments autorisés et recommandés par l' OMS et le PNLP au Mali; et n'implique pas de nouvelles méthodes. Les risques pour les participants sont limités à ceux qui sont associés à l'utilisation de la SP + AQ et le prélèvement de sang. Risques associés à la piqûre des doigts et au talon ainsi que le prélèvement veineux qui sont douloureux et souvent associés et des ecchymoses sur le site de la piqûre, et rarement une infection. L'utilisation de la SP + AQ peut être associée à des effets secondaires de ces médicaments, y compris les allergies. Les sujets présentant une allergie connue aux médicaments de l'étude seront retirés de l'étude. Plus de 800 000 doses de SP et AQ ont été données pour la CPS sans report d'événements indésirables graves associés au médicament, y compris le renforcement de la surveillance [9 ; 14].

Cependant, un danger potentiel de l'intervention est l'impact possible de l'administration du médicament à grande échelle sur deux ou trois ans sur la sensibilité des parasites aux médicaments antipaludiques. La possibilité que la CPS avec SP + AQ pourrait induire une résistance à ces médicaments chez P. *falciparum* a été étudiée dans un certain nombre d'essais de la CPS. La sélection des parasites portant des mutations qui confèrent une résistance à la pyriméthamine ou la sulfadoxine a été démontrée dans certaines études mais pas dans d'autres [9]. Toutefois, en raison de la prévalence de la parasitémie chez les enfants qui avaient reçu la CPS était sensiblement inférieure à celle du groupe témoin, le nombre total de parasites portant des marqueurs de résistance était inférieure chez les enfants qui avaient reçu la CPS que chez les enfants dans le groupe contrôle. La SP a été largement utilisée dans le traitement préventif intermittent chez la femme enceinte et n'a pas été associée à une accélération de la résistance à la SP en Afrique de l'Ouest. De même, l'utilisation de la SP dans la mise en œuvre pilote au Mali n'a pas été associée à une augmentation de la fréquence des marqueurs moléculaires de la résistance de *P. falciparum* à la SP [13]. En outre, le risque potentiel de la CPS avec SP + AQ dans l'induction de la résistance des parasites du paludisme a été soigneusement examiné par le groupe d'experts techniques de l'OMS et considéré comme un risque acceptable à la lumière des principaux avantages de l'intervention. Les échantillons recueillis sur du papier filtre seront analysés pour les marqueurs de la résistance à la SP et AQ avant et deux ans après pour suivre l'impact de la CPS sur sensibilité des parasites du paludisme aux médicaments de l'étude, conformément aux recommandations de l'OMS.

La conduite de l'essai n'imposera pas de coûts supplémentaires sur les services de santé locaux. Le projet contribuera aux coûts supplémentaires liés à l'étude ainsi que le renforcement du système de santé dans les zones d'étude.

6.5 **Evénements indésirables**

Les risques liés à la participation à l'étude sont mineurs. Dans cette étude, on utilisera uniquement des produits autorisés et on n'utilisera pas de nouvelles méthodes. Plus de 800 000 doses de traitement de SP et AQ ont été administrées dans le cadre de la CPS sans qu'aucun événement indésirable grave (EIG) soit associé au traitement, y compris lorsque la surveillance renforcée a été utilisée [8 ; 13]. Néanmoins les effets indésirables graves seront surveillés tout au long de la période d'étude et tout EIG lié aux médicaments de l'étude sera rapporté au Comité d'Ethique de la Faculté de Médecine, de Pharmacie et d'Odontostomatologie de l'Université des Sciences des Techniques et Technologie de Bamako dans les 48 heures après que l'investigateur principal aura été mis au courant.

Définitions et procédures d'enregistrement et de déclaration des événements indésirables (EI) et les événements indésirables graves (EIG)

Un événement indésirable (EI) est défini comme tout symptôme clinique ou un signe qui se produit chez les enfants de l'étude après l'administration des médicaments de l'étude qui peuvent ou peuvent ne pas avoir une relation de cause à effet avec les médicaments de l'étude. Un événement indésirable grave (EIG) est une condition clinique qui répond à au moins un des critères suivants : entraîner la mort, mettre en danger la vie (l'enfant était en danger de mort au moment de l'événement indésirable), provoque un handicap / incapacité. Les EI seront classés par ordre de gravité, la causalité et le devenir comme décrits ci-dessous.

Gravité. La gravité d'un événement indésirable clinique doit être marquée selon le barème suivant :

• Léger: prise de conscience de signes ou symptômes, mais facilement tolérés.

• Modéré: assez pour causer des interférences avec l'activité habituelle, inconfort.

• Sévère : invalidant avec impossibilité d'exercer une activité habituelle.

• Menace le pronostic vital : Les patients à risque de mort au moment de l'événement.

• Décès

Évaluation de la causalité et le devenir. La relation entre les médicaments de l'étude et la réalisation de chaque EI / EIG sera déterminée sur la base de leur jugement clinique. D'autres causes, comme l'histoire naturelle des maladies sous-jacentes, un traitement concomitant, d'autres facteurs de risque et la relation temporelle de l'événement pour le médicament de l'étude seront pris en compte et étudiés. La relation d'un événement indésirable au médicament d'étude doit être évaluée selon les définitions suivantes:

• Définitivement non lié : les événements qui ont eu lieu avant l'administration des médicaments à l'étude ou à des événements qui n'ont évidemment rien à voir avec l'étude (une blessure accidentelle par exemple).

• Peu probable : Il n'existe aucune association temporelle raisonnable entre le médicament de l'étude et l'événement suspect et l'événement aurait pu être produit par l'état clinique de l'enfant ou d'autres médicaments concomitants.

• Possible : L'événement indésirable peut ou peut ne pas avoir une association temporelle raisonnable à l'administration du médicament de l'étude, mais la nature de l'événement est telle qu'une association avec le médicament de l'étude ne peut pas être exclue. L'événement pourrait être lié à l'état clinique de l'enfant ou aux médicaments concomitants.

• Probable: L'événement indésirable suit une séquence temporelle raisonnable après l'administration de médicaments à l'étude, se calme à l'arrêt du médicament, et ne peut pas être raisonnablement expliqué par l'état clinique connu de l'enfant.
• Définitivement liés : les événements qui n'ont pas d'incertitude dans leur association à l'administration de médicaments à l'étude.

Devenir. Le devenir de chaque EI sera évalué selon la classification suivante :
• Complètement guéri : L'enfant a complètement récupéré sans effets résiduels observables.
• Pas encore complètement guéri: l'état de l'enfant s'est amélioré, mais il a encore quelques effets résiduels
• Détérioration : l'état général de l'enfant a empiré
• Des dommages permanents : l'EI a entraîné une déficience permanente
• Décès: l'enfant est mort à cause de l'EI
• En cours: l'EI reste le même qu'au début
• Inconnu: le devenir de l'EI n'est pas connu en raison du fait qu'il est perdu de vue.

6.6 Avantages potentiels

Les avantages de la participation à l'étude comprennent la protection contre le paludisme clinique conférée par la CPS et l'amélioration de la supervision des soins de santé fournis par l'ASC et le personnel de santé. Trouver la méthode de distribution optimale de la CPS permettra un déploiement optimal de la stratégie à travers le pays pour un impact plus important sur la morbidité et la mortalité palustre au Mali et dans d'autres pays. L'étude devra également fournir des informations sur l'acquisition de l'immunité contre le paludisme et peut indiquer si l'arrêt à long terme de la CPS n'augmentera pas le risque de paludisme ultérieur et justifier des mesures de contrôle supplémentaires.

6.7 Confidentialité

L'anonymat du sujet sera maintenu. Les participants ne seront pas identifiés dans les rapports rendus publics de cette étude. Tous les dossiers seront gardés confidentiels dans la mesure prévue par les lois et règlementations. Les moniteurs de l'étude et d'autres représentants légaux des autorités réglementaires pourront inspecter tous les documents et les dossiers qui doivent être conservés par l'investigateur. Tous les échantillons, les formulaires d'évaluation, les rapports et autres documents qui quittent le site ne seront identifiés que par un numéro de code afin de maintenir la confidentialité.

6.8 Mineurs ou sujets souffrant de troubles mentaux.

Les sujets mineurs (de moins de 5 ans) avec ou sans troubles mentaux seront inclus dans cette étude après obtention du consentement éclairé par le parent ou le tuteur.

7. Fin de l'Étude

La mise en œuvre de la CPS a déjà commencé dans le district de Koutiala au Mali ainsi que dans quatre autres districts. Le plan est de l'étendre à d'autres districts chaque année. Il est prévu que d'ici la fin de ce projet en 2017, la mise en œuvre de la CPS sera étendue sur l'ensemble du pays, y compris le district de Ouéléssébougou. PMI est un des principaux partenaires du Mali qui soutiennent la mise en œuvre de la CPS au Mali. Si l'extension de la mise en œuvre de la CPS est retardée, les investigateurs travailleront avec le PNLP et le PMI pour s'assurer que la mise en œuvre de la CPS utilisant l'approche optimale continue à Ouéléssébougou à la fin de cette étude.

8. Plan de monitorage et de gestion des déchets

Le MRTC conduit la recherche clinique avec le NIAID / NIH depuis plus de 20 ans. A travers cette collaboration, le MRTC est actuellement doté d'un laboratoire clinique certifié par le Collège des Pathologistes Américains (CAP). Le personnel est bien au courant et formé sur la gestion et le suivi des déchets. La gestion et le suivi des déchets seront effectués selon les POS à l'annexe 2. Les déchets principaux associés à l'étude comprennent les déchets biohazard (solides et liquides). Les cliniciens et le personnel de laboratoire sont responsables des poubelles de sécurité, des déchets biologiques dangereux selon la POS. Cette responsabilité comprend le tri des déchets non dangereux (comme le papier) de déchets dangereux pour minimiser l'impact sur l'environnement. Les laborantins et cliniciens reçoivent des vêtements de protection tels que des blouses de laboratoire et des gants ainsi que du matériel pour collecter les déchets à risque biologique. Les locaux du programme IMEP à Ouéléssébougou comportent un incinérateur pour le traitement des déchets solides. Les déchets produits par les cliniciens dans les villages seront traités localement si un incinérateur est disponible ou seront transportés au laboratoire central de Ouéléssébougou. Les sites équipés d'incinérateurs serviront de sites d'élimination finale.. Comme toutes les procédures cliniques, de laboratoire et des données, le respect des SOP est surveillé sur une base régulière par les chercheurs principaux du projet.

9. Développement des capacités

Le projet renforcera la capacité du système de santé local ainsi qu'au MRTC. Le personnel du centre de santé des villages sélectionnés et au niveau des districts seront formés pour effectuer les interventions de la CPS avec le soutien de l'équipe du projet composée de MRTC, LMIV et PNLP. Des outils de mise en œuvre, de suivi et d'évaluation seront élaborés et pourraient être utilisés dans d'autres districts. Les cas de paludisme seront correctement diagnostiqués, traités et déclarés. Le système de surveillance intégré des maladies sera renforcé dans les localités sélectionnées. Des jeunes scientifiques au MRTC travailleront sur ce projet dans le cadre de la formation continue et le renforcement des capacités du MRTC. Le projet contribuera à acquérir des équipements cliniques, de laboratoire et informatique et serviront à entretenir le matériel existant qui sera utilisé pour le projet au MRTC. Les données recueillies seront utilisées pour des mémoires de Master et des thèses de doctorat à l'Université de Bamako et ailleurs.

10. Plan de publication des données et le transfert des connaissances

Les résultats de l'étude seront présentés et discutés au cours des réunions régulières du comité de gestion du projet (CGP), comprenant un représentant du PNLP et un de PMI qui seront établis pour ce projet. Chaque année, les résultats de l'étude seront présentés dans des communautés où les essais ont été effectués et les autorités sanitaires locales et nationales à travers des réunions ad hoc, des ateliers de présentation des données et / ou au cours de la célébration de la journée africaine du paludisme au Mali. Les résultats de l'étude seront également présentés lors de réunions scientifiques nationales et internationales, y compris les réunions sur la lutte contre le paludisme ainsi que des publications dans des revues professionnelles. Comme dans les études précédentes sur la CPS, des rapports détaillés et des publications scientifiques seront soumis au Programme Mondial de lutte contre le Paludisme de l'OMS. Les résultats seront également partagés régulièrement avec les représentations locales de l'OMS et de l'UNICEF ainsi que d'autres partenaires au Mali par le biais de réunions régulières de " Health cluster", qui ont lieu régulièrement au bureau de l'OMS à Bamako et qui impliquent des représentants du Ministère de la Santé, l'OMS, l'UNICEF, l'USAID et d'autres partenaires de la santé. Les résultats sous forme de rapport seront transmis aussi au comité d'éthique de la FMPOS.

11. Chronologie détaillée

Le calendrier des activités clés de l'étude est résumé dans le tableau 1.

Tableau 1. Les activités principales de l'étude et la chronologie

c

	Mois																																			
**Activités**	1*	2	3	4	5	6	7	8	9	10	11	12	13	14	15	16	17	18	19	20	21	22	23	24	25	26	27	28	29	30	31	32	33	34	35	36
Finalisation du protocole, SOP, CRFs	■	■																																		
soumission du protocole au CE		■	■																																	
Sélection des airs de sante et la randomisation				■																																
Formation des personnels de l'étude				■	■																															
recensement				■	■																															
Enquête transversale (commence au début de la saison)					■	■																														
Administration de la CPS						■	■	■										■	■	■	■									■	■	■	■			
La surveillance passive						■	■	■	■	■	■	■	■	■	■	■	■	■	■	■	■	■	■	■	■	■	■	■	■	■	■	■	■	■		
Fin de l'enquête transversale de la saison									■	■											■	■											■	■		
Le développement et le test de la base de données				■	■	■																														
La gestion des données							■	■	■	■	■	■	■	■	■	■	■	■	■	■	■	■	■	■	■	■	■	■	■	■	■	■	■	■		
L'analyse des données et rapports											■	■											■	■											■	■

* Mars 2014

Références

1. World Health Organization (2012). World Malaria Report 2012 Available: http://www.who.int/malaria/publications/world_malaria_report_2012/en/index.html

2. Lengeler C. Insecticide-treated bed nets and curtains for preventing malaria. Cochrane Database Syst Rev. 2004 ;(2):CD000363.

3. Dicko A, Diallo AI, Tembine I et al. Intermittent preventive treatment of malaria provides substantial protection against malaria in children already protected by an insecticide-treated bednet in Mali: a randomised, double-blind, placebo-controlled trial. PLoS Med 2011; 8: 1000407.

4. World Health Organization WHO Global Malaria Programme (2012). WHO policy recommendation: Seasonal malaria chemoprevention (SMC) for Plasmodium falciparum malaria control in highly seasonal transmission areas of the Sahel sub-region in Africa. March 2012. Available at: http://www.who.int/malaria/publications/atoz/who_smc_policy_recommendation/en/

5. World Health Organization (2013). Seasonal malaria chemoprevention with sulfadoxine-pyrimethamine plus amodiaquine in children: A field guide. Accessible at http://www.who.int/malaria/publications/atoz/9789241504737/en/index.html

6. Kweku M, Webster J, Adjuik M. et al. Options for the delivery of intermittent preventive treatment for malaria to children: a community randomised trial. PLoS One 2009; 4: e7256.

7. Bojang KA, Akor F, Conteh L, et al. Two strategies for the delivery of IPTc in an area of seasonal malaria transmission in The Gambia: a randomised controlled trial. PLoS Medicine. 2011; 8:e1000409.

8. Konate AT, Yaro JB, Ouedraogo AZ et al. Intermittent preventive treatment of malaria provides substantial protection against malaria in children already protected by an insecticide-treated bednet in Burkina Faso : a randomised, double-blind, placebo-controlled trial. PLoS Med 2011; 8: e1000408.

9. Wilson AL. A systematic review and meta-analysis of the efficacy and safety of intermittent preventive treatment in children (IPTc). Plos One 2011; 6: e16976.

10. World Health Organization (2000). Severe falciparum malaria. Trans R Soc Trop Med Hyg 94: S1–S90.

11. Plowe CV, Djimde A, Bouare M, Doumbo O, Wellems TE (1995). Pyrimethamine and proguanil resistance-conferring mutations in Plasmodium falciparum dihydrofolate reductase: polymerase chain reaction methods for surveillance in Africa. Am J Trop Med Hyg 52: 565–568.

12. Djimde´ A, Doumbo OK, Cortese JF, Kayentao K, Doumbo S, et al. (2001) A molecular marker for chloroquine-resistant falciparum malaria. N Engl J Med 344: 257–263.

13. Dicko A, Sagara I, Djimde´ AA, Toure´ SO, Traore M, et al. (2010) Molecular markers of resistance to sulphadoxine-pyrimethamine one year after implementation of intermittent preventive treatment of malaria in infants in Mali. Malar J 9: 9.

14. Ndiaye JL, Cissé B, Ba EH et al. Safety of seasonal intermittent preventive treatment against malaria with sulfadoxine pyrimethamine + amodiaquine when delivered to children under 10 years of age by district health staff in Senegal. PLoS Med: submitted.

15. Dicko A, Toure SO, Traore M, et a,. Increase in EPI vaccines coverage after implementation of intermittent preventive treatment of malaria in infant with Sulfadoxine -pyrimethamine in the district of Kolokani, Mali: results from a cluster randomized control trial. BMC Public Health. 2011 Jul 18;11:573.

16. Sagara I, Dicko A, Ellis RD, et al. A randomized controlled phase 2 trial of the blood stage AMA1-C1/Alhydrogel malaria vaccine in children in Mali. Vaccine. 2009 May 18; 27(23):3090-8.
